# Mutant GNAS drives a pyloric metaplasia with tumor suppressive glycans in intraductal papillary mucinous neoplasia

**DOI:** 10.1016/j.celrep.2025.116684

**Published:** 2025-12-09

**Authors:** Vincent Quoc-Huy Trinh, Katherine E. Ankenbauer, Sabrina M. Torbit, Christopher P. Taranto, Jiayue Liu, Maelle Batardiere, Bhoj Kumar, H. Carlo Maurer, Frank Revetta, Zhengyi Chen, Angela R.S. Kruse, Audra M. Judd, Celina Copeland, Jahg Wong, Olivia Ben-Levy, Brenda Jarvis, Monica Brown, Jeffrey W. Brown, Koushik Das, Yuki Makino, Jeffrey M. Spraggins, Ken S. Lau, Parastoo Azadi, Anirban Maitra, Marcus C.B. Tan, Kathleen E. DelGiorno

**Affiliations:** 1 Department of Surgery, Vanderbilt University Medical Center, Nashville, TN, USA; 2 Institute for Research in Immunology and Cancer and the Centre de recherche du Centre hospitalier de l’Université de Montréal, University of Montreal, Montreal, QC, Canada; 3 Department of Cell and Developmental Biology, Vanderbilt University, Nashville, TN, USA; 4 Program in Cancer Biology, Vanderbilt University, Nashville, TN, USA; 5 Complex Carbohydrate Research Center, University of Georgia, Athens, GA, USA; 6 Department of Internal Medicine II, Klinikum Rechts der Isar, School of Medicine, Technical University of Munich, München, Germany; 7 Department of Pathology, Microbiology and Immunology, Vanderbilt University Medical Center, Nashville, TN, USA; 8 Epithelial Biology Center, Vanderbilt University Medical Center, Nashville, TN 37232, USA; 9 Center for Computational Systems Biology, Vanderbilt University, Nashville, TN 37232, USA; 10 Chemical and Physical Biology Program, Vanderbilt University, Nashville, TN 37232, USA; 11 Mass Spectrometry Research Center, Vanderbilt University School of Medicine, Nashville, TN, USA; 12 Division of Gastroenterology, Department of Medicine, Washington University in St. Louis, St. Louis, MO, USA; 13 Department of Translational Molecular Pathology, The University of Texas MD Anderson Cancer Center, Houston, TX, USA; 14 Sheikh Ahmed Pancreatic Cancer Research Center, The University of Texas MD Anderson Cancer Center, Houston, TX, USA; 15 Department of Biochemistry, Vanderbilt University School of Medicine, Nashville, TN, USA; 16 Department of Chemistry, Vanderbilt University, Nashville, TN, USA; 17 Department of Pathology, Microbiology and Immunology, Vanderbilt University Medical Center, Nashville, TN, USA; 18 Vanderbilt Ingram Cancer Center, Vanderbilt University Medical Center, Nashville, TN, USA; 19 Vanderbilt Digestive Disease Research Center, Vanderbilt University Medical Center, Nashville, TN, USA; 20 These authors contributed equally; 21 Lead contact

## Abstract

Intraductal papillary mucinous neoplasms (IPMNs) are cystic lesions and bona fide precursors of pancreatic ductal adenocarcinoma (PDAC), one of the deadliest solid tumors. Although ~90% of IPMNs are detected before PDAC forms, markers distinguishing benign from malignant disease are lacking, resulting in an abundance of unnecessary, invasive surgeries. Recent studies show that pancreatic precancer assumes a pyloric phenotype. To identify the regulators of this plasticity, cell lines, organoids, tumors from mouse models of IPMNs, and patient samples underwent multiplex immunostaining, RNA sequencing, glycosylation profiling, and computational analysis. These data revealed that GNAS^R201C^ drives an indolent phenotype in IPMNs by amplifying a differentiated, pyloric phenotype through SPDEF/CREB3L1, which is characterized by distinct glycans. Acting as a glycan rheostat, GNAS^R201C^ elevates LacdiNAcs at the expense of pro-tumorigenic acidic Lewis epitopes, inhibiting cancer cell invasion and disease progression. LacdiNAcs and 3′-sulfo-Le^A/C^ are mutually exclusive and may serve as markers to risk stratify IPMN patients for surgery.

## In brief

Trinh et al. show that mutant GNAS promotes pyloric-type differentiation and reprograms glycosylation in pancreatic cystic precancerous lesions. This indolent program slows progression, and glycan signatures distinguish low- from high-grade IPMN.

## Graphical Abstract

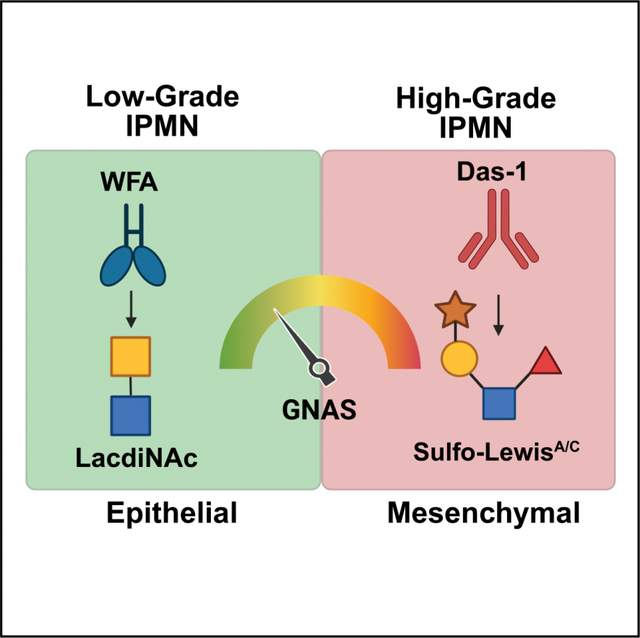

## INTRODUCTION

Pancreatic ductal adenocarcinoma (PDAC) is currently the third leading cause of cancer-related deaths in the United States and is slated to become second by the year 2040.^1^ This is largely due to late detection—pancreatic tumors grow silently, approximately 85% of patients present with incurable locally advanced or metastatic disease, and distant metastatic spread can occur when the tumors are small (<5 mm). On the other hand, the time required for PDAC to emerge from a normal cell is long, some 15–20 years.^2^ Thus, improving our understanding of the early events in neoplastic transformation is necessary and crucial to allow earlier diagnosis of PDAC and also important therapeutically to gain insights into how progression to cancer can be blocked or even reversed.

A major obstacle to the study of early events in pancreatic carcinogenesis is that the main precursor to PDAC, pancreatic intraepithelial neoplasia (PanIN), is clinically silent and radiographically occult. Intriguingly, the opposite is true of intraductal papillary mucinous neoplasms (IPMNs), which contribute to approximately 25% of cases of PDACs.^3^ As IPMNs are cystic rather than microscopic like PanIN, they are easily identified on abdominal imaging scans, and as a result, 90% are diagnosed before cancer presents. Thus, for most IPMNs, there is a window of opportunity for surveillance and intervention before invasive disease develops.^4^ For all pre-malignant tumors, accurate assessment of the risk of malignant transformation is essential for clinical decision-making. This is particularly true of IPMNs, because the risks of both under- and over-treatment are high. If cancer risk is under-estimated and the patient is surveilled, then the emergence of PDAC could be lethal. Conversely, surgery for IPMNs carries with it very significant risks—the postoperative complication rate is 30%–50%, and the 90-day mortality is 3–5%.^5^ Unfortunately, markers distinguishing low- and high-grade IPMNs are currently lacking, resulting in an abundance of unnecessary surgeries. Approximately 50% of patients undergoing surgery for IPMNs are found to lack PDAC upon final pathology. Therefore, better markers are desperately needed for the risk stratification of IPMN patients.

Oncogenic mutations in *KRAS* are common in human PanIN lesions and have been shown to drive tumorigenesis through increasingly dysplastic grades of PanIN to PDAC in genetically engineered mouse models (GEMMs).^6^ Like PanIN, IPMNs harbor *KRAS* mutations (~80%), but, distinct from PanIN, approximately two-thirds also express oncogenic GNAS (~66%) driver mutations.^7^ In GEMMs, combined expression of both mutant *KRAS* and *GNAS* has been shown to drive PDAC formation through a mixed phenotype of PanIN and IPMNs.^8^ In cell lines, *GNAS*^*R201H/C*^ mutations have been shown to drive an indolent, less-aggressive phenotype, potentially by limiting *KRAS* signaling.^8,9^ Recent studies, however, have shown that high-grade human IPMNs bearing *GNAS* mutations are accompanied by a loss of the wild-type allele, suggesting that allelic imbalance drives disease. Further, these IPMNs are accompanied by additional mutations driving MAPK signaling and tumor-suppressor loss; *GNAS* mutations alone are likely insufficient to drive IPMN progression.^10,11^

Metaplasia is an initiating event in both PanIN and IPMN formation. Metaplasia is a pathological term for the transdifferentiation of one cell type to another and is a form of plasticity common to injury- and oncogene-induced disease progression in the gastrointestinal tract.^12^ While it is largely thought to represent a reactive tissue response to mitigate injury, it is also considered to be the first step in tumorigenesis in several organs. Metaplasia falling under the general rubric of pyloric-like has been reported in stomach injury and tumorigenesis, serrated colorectal polyps, and pancreatic injury and tumorigenesis occurring through PanIN progression.^13–16^ Metaplasia in the stomach, or spasmolytic polypeptide-expressing metaplasia (SPEM), is the best characterized and reflects the transition of gastric chief cells (digestive enzyme-producing cells) to a phenotype described by the expression of specific markers (e.g., MUC6, TFF2, AQP5, CD44v9, and GKN3).^17^ When SPEM is accompanied by the presence of a foveolar pit cell lineage (TFF1, GKN1, GKN2, and MUC5AC), the phenotype is a pyloric metaplasia due to a recapitulation of the pylorus region of the stomach.^13,17^ Recently, we combined single-cell RNA-sequencing (scRNA-seq), electron microscopy, and histopathology and showed that injury-induced acinar-to-ductal metaplasia (ADM) in the pancreas results in the formation of chemosensory tuft cells, hormone-producing enteroendocrine cells, and a population bearing canonical markers of SPEM.^13^ ADM and PanIN resulting from oncogenic *Kras*^*G12D*^ expression are also characterized by SPEM with the additional formation of a separate, distinct foveolar pit cell-like population, reflecting pyloric metaplasia.^13,14^ The functional role of individual pyloric markers has previously been studied in GEMMs of gastric disease; however, the program itself is hypothesized to represent a shared mechanism by which gastrointestinal organs respond to injury and tumorigenesis.^17^

Here, we assayed patient IPMN and murine models for the expression of pyloric markers. We show that mutant *GNAS* is sufficient to amplify a pyloric phenotype, mucus production, and distinct glycosylation changes and identify transcription factors SPDEF and CREB3L1 as critical regulators of this program. Finally, we show that *GNAS*^*R201C*^-induced glycan changes contribute to an indolent phenotype and can distinguish high-from low-grade IPMNs to risk stratify IPMN patients.

## RESULTS

### Human IPMNs recapitulate pyloric metaplasia

**Figure 1.**
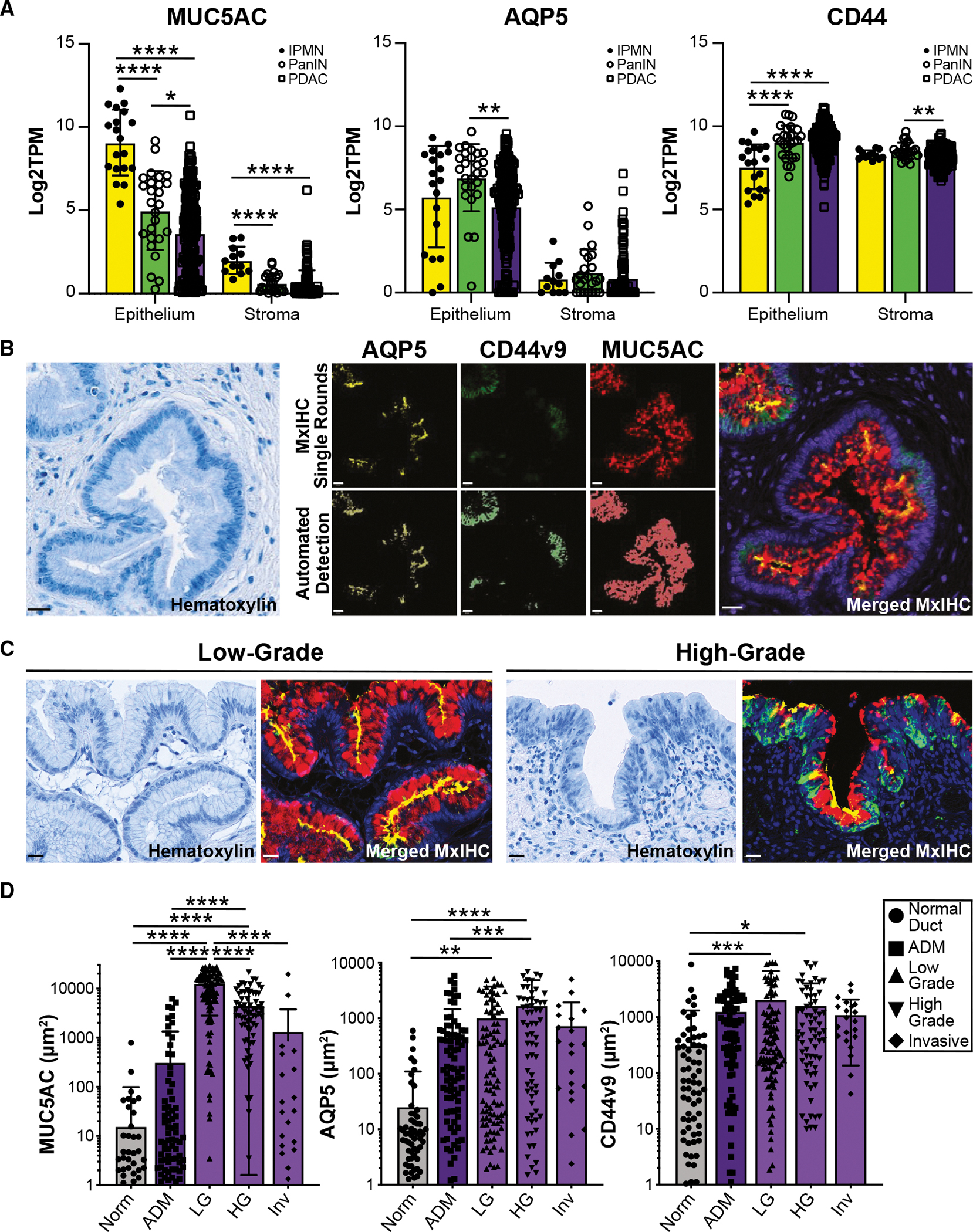
Human IPMN are characterized by pyloric metaplasia (A) Bar plots comparing the expression of *MUC5AC*, *AQP5*, or *CD44* in the epithelium and stroma in IPMNs (yellow, *n* = 19), PanIN (green, *n* = 26), and PDAC (purple, *n* = 197 epithelium, 124 stroma).^19^ (B) Hematoxylin staining and pseudo-colored immunohistochemical staining for MUC5AC (red), AQP5 (yellow), or CD44v9 (green), top row, and automated detection of signal (bottom row) by QuPath to merge MxIHC data. ^21^Scale bars, 20 μm. (C) Examples of hematoxylin staining or merged MxIHC of low-grade (left) or high-grade (right) IPMNs. Scale bars, 20 μm. (D) Quantification of staining in (B)–(C) for 40 IPMN patients, including normal ducts (ND; *n* = 95–109), acinar-to-ductal metaplasia (ADM; *n* = 109), low-grade IPMN (LG; *n* = 110), high-grade IPMN (HG; *n* = 70), and invasive IPMN (Inv; *n* = 22). *p < 0.05; **p < 0.01; ***p < 0.005; and ****p < 0.001.

Metaplastic tissues bearing markers of the gastric pylorus have been reported in injury and tumorigenesis in several gastrointestinal organs.^13,15,17^ To evaluate the expression of pyloric metaplasia markers in IPMNs, we examined the expression of *MUC5AC* (pit lineage marker), *TFF2*, *AQP5*, and *CD44* (SPEM markers), which produces the splicing variant CD44v9, in published RNA-seq datasets derived from patient samples. Previously, Bernard et al. generated an scRNA-seq dataset composed of epithelium and stroma from either low- or high-grade IPMN (*n* = 2 each) or IPMN-associated PDAC (*n* = 2; Figures S1A–S1C).^18^ Analysis of this dataset for molecular markers of the IPMN subtype revealed widespread expression of *MUC5AC* throughout the epithelium of all samples, with *MUC6* and *MUC2* differentiating gastric from intestinal-type IPMNs, respectively (Figure S1D). Expression of *TFF2* and *AQP5* was enriched in the epithelium, whereas *CD44* was identified in both the epithelium and stroma (Figure S1E). To evaluate a second dataset, we interrogated bulk RNA-seq generated by Maurer et al. of laser capture-dissected epithelium or adjacent stroma from patient IPMNs (*n* = 19), PanIN (*n* = 26), or PDAC (*n* = 197 epithelium, 124 stroma).^19,20^ Like our analysis of IPMN scRNA-seq data, we identified the expression of *MUC5AC* and *AQP5*, enriched in the epithelium of all disease states, and *CD44*, in both epithelial and stromal populations (Figure 1A). Finally, we examined the expression of an SPEM gene signature generated from our previously published murine pancreatitis scRNA-seq dataset in the Maurer dataset.^20^ We identified the enrichment of several additional markers in IPMNs, as compared to PDAC (Figure S2).

To confirm the expression of pyloric metaplasia markers in patient IPMN samples, we conducted multiplex immunohistochemistry (MxIHC) on a cohort of 40 patients (Table S1). Serial staining was conducted for MUC5AC, AQP5, and CD44v9, and expression was scored in multiple tissue compartments within each sample. Signal for each stain was automatically detected using QuPath and overlaid to generate merged images (Figure 1B).^21^ Altogether, expression was evaluated in normal ducts (Norm; *n* = 95–109 regions of interest [ROIs]), ADM (*n* = 109 ROIs), low-grade IPMNs (LG; *n* = 110 ROIs), high-grade IPMN (HG; *n* = 70 ROIs), and foci of invasive PDAC (Inv; *n* = 22 ROIs). All markers were detected; interestingly, protein localization changed between low- and high-grade IPMNs (Figure 1C). Consistent with previous reports, we identified a significant increase in MUC5AC between normal ducts and IPMNs and a loss of expression with progression from low-grade to invasive IPMNs.^18^ Expression of both AQP5 and CD44v9 increased with disease progression, reaching significance in low- and high-grade IPMN (Figures 1D, S3A, and S3B). Stratifying IPMNs by molecular subtype (gastric, intestinal, and pancreatobiliary-like), we identified pyloric metaplasia in all subtypes, with no significant difference in the expression of MUC5AC or AQP5 between gastric and intestinal-type IPMNs (Figure S3C). Collectively, these data demonstrate that a significant increase in pyloric metaplasia accompanies IPMN formation in patients.

### Mutant GNAS drives a pyloric phenotype

**Figure 2.**
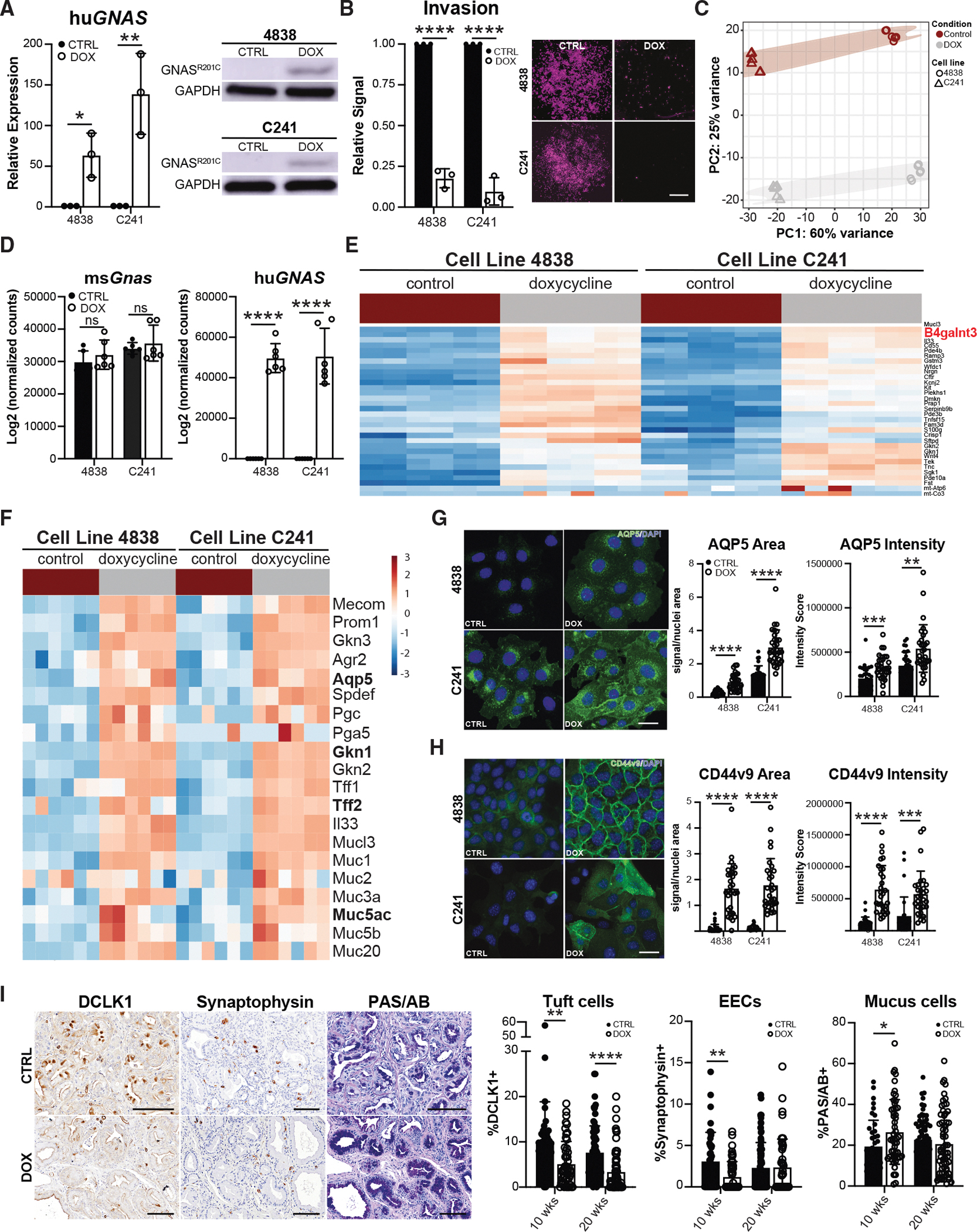
Mutant GNAS drives a pyloric metaplasia program (A) RT-qPCR (*n* = 3) or western blot for human *GNAS* (hu*GNAS*) in 4838 and C241 cell lines treated ± doxycycline (DOX). (B) Representative images and quantification of invasion for both cell lines treated ± DOX. Scale bar, 1 mm. (C) Principal component analysis (PCA) of RNA-seq conducted on both cell lines ± DOX (*n* = 6, 3 biological replicates per line). (D) Bar plots of murine (ms) or human (hu) *GNAS* expression, determined by RNA-seq, in both cell lines. (E) Heatmap of top upregulated genes in both cell lines with DOX. (F) Heatmap of pyloric metaplasia marker gene expression in both cell lines ± DOX. (G and H) (G) Immunofluorescence and quantification of signal area and intensity for AQP5 and (H) CD44v9 in both cell lines ± DOX (*n* = 3 biological replicates, 10 ROIs per replicate). Scale bars, 25 μm. (I) IHC and quantification of tuft marker DCLK1, EEC marker synaptophysin, or mucin staining by PAS/AB in *Kras*;*GNAS* pancreata from mice fed a control or DOX diet (*n* = 6 mice/condition). Scale bars, 100 μm. *p < 0.05; **p < 0.01; ***p < 0.005; and ****p < 0.001.

While PanIN and IPMNs have both been shown to express oncogenic *KRAS*, most IPMNs also express mutant *GNAS*.^7^ Previous studies have shown that *GNAS*^*R201C/H*^ mutations inhibit PDAC aggressiveness.^8,9^ To determine if *GNAS*^*R201C*^ drives indolence by amplifying a pyloric phenotype, we used two cell lines (4838 and C241) generated from murine IPMN-derived PDAC harboring a *Kras*^*G12D*^ mutation that conditionally express human *GNAS*^*R201C*^ upon doxycycline (DOX) treatment (Figure 2A).^8,22^ Consistent with previous studies, we identified a significant decrease in cancer cell invasion in both cell lines with DOX induction of *GNAS*^*R201C*^ (Figure 2B). To identify potential mechanisms by which *GNAS*^*R201C*^ inhibits tumor cell aggressiveness, both cell lines were treated ± DOX and underwent RNA sequencing (*n* = 6, 3 biological replicates/condition; Figures 2C–2F and S4; Tables S2 and S3). As expected, there was a significant increase in human (hu*GNAS*), but not murine *Gnas* (mu*Gnas*), expression (Figure 2D). Significant differences were identified between the two cell lines, likely due to biological heterogeneity or differences in tumor-suppressor mutations (Figures 2E, 2F, and S4); however, both cell lines showed an increase in the expression of pyloric markers (*Gkn1*, *Aqp5*, and *Tff2*) and mucin genes (*Muc5ac*) with induction of *GNAS*^*R201C*^ expression (Figure 2F). Correspondingly, we saw an increase in the expression of epithelial markers (*Epcam* and *Cldn2*) and a decrease in mesenchymal markers (*Fn1* and *Zeb1*; Figure S4) by quantitative reverse-transcriptase PCR (RT-qPCR), suggesting a mesenchymal-to-epithelial transition (MET). Consistent with our assessment, gene set enrichment analysis (GSEA) identified a decrease in the expression of genes associated with EMT and EGFR signaling, as well as an increase in the expression of genes associated with early gastric cancer (Figures S4F and S4G).

To confirm gene expression changes for select markers at the protein level, we conducted immunofluorescence (IF) on 4838 and C241 cells ± DOX. As shown in Figures 2G and 2H, we saw a significant increase in the overall expression and intensity of both AQP5 and CD44v9. Mass spectrometry was also performed to validate changes in select protein expression (Figures S5A and S5B). To determine whether *GNAS*^*R201C*^ preferentially drives mucus cell production *in vivo*, we next evaluated cell-type abundance in *Kras*;*GNAS* mice. Pancreata were collected from *Kras*;*GNAS* mice treated ± DOX for either 10 or 20 weeks (*n* = 3 mice/condition).^8^ H&E staining was performed to evaluate disease progression and cyst formation. The two groups were found to be equivalent in terms of lesion grade; however, DOX treatment was found to enhance cyst formation (Figure S5C), as previously described.^8^ Regions of cysts/IPMN and PanIN lesions were then identified by H&E (~3 regions of each/slide), with most mice harboring both. Previously, we showed that metaplasia in the pancreas results in the *de novo* formation of several differentiated cell types, such as tuft, enteroendocrine (EEC), and mucus-producing cells.^13,23–25^ Serial sections were stained for tuft cell marker DCLK1,^23^ EEC marker synaptophysin,^26^ and mucins (PAS/AB) and then quantified. As shown in Figure 2I, *GNAS*^*R201C*^ expression was sufficient to significantly decrease tuft (9.9%, 45 lesions vs. 5.2%, 52 lesions) and enteroendocrine cell numbers (3.1%, 42 lesions vs. 1.2%, 52 lesions), while significantly increasing mucin production (19.6%, 40 lesions vs. 26.6%, 52 lesions) in low-grade lesions, consistent with our analysis of human data (Figure S5D; Table S4). Collectively, these data demonstrate that *GNAS*^*R201C*^ shifts cells away from aggressive behavior and toward a mucinous, pyloric phenotype, potentially limiting tumor cell aggressiveness by driving a program of differentiation.

### Identification of master regulators of pyloric metaplasia

**Figure 3.**
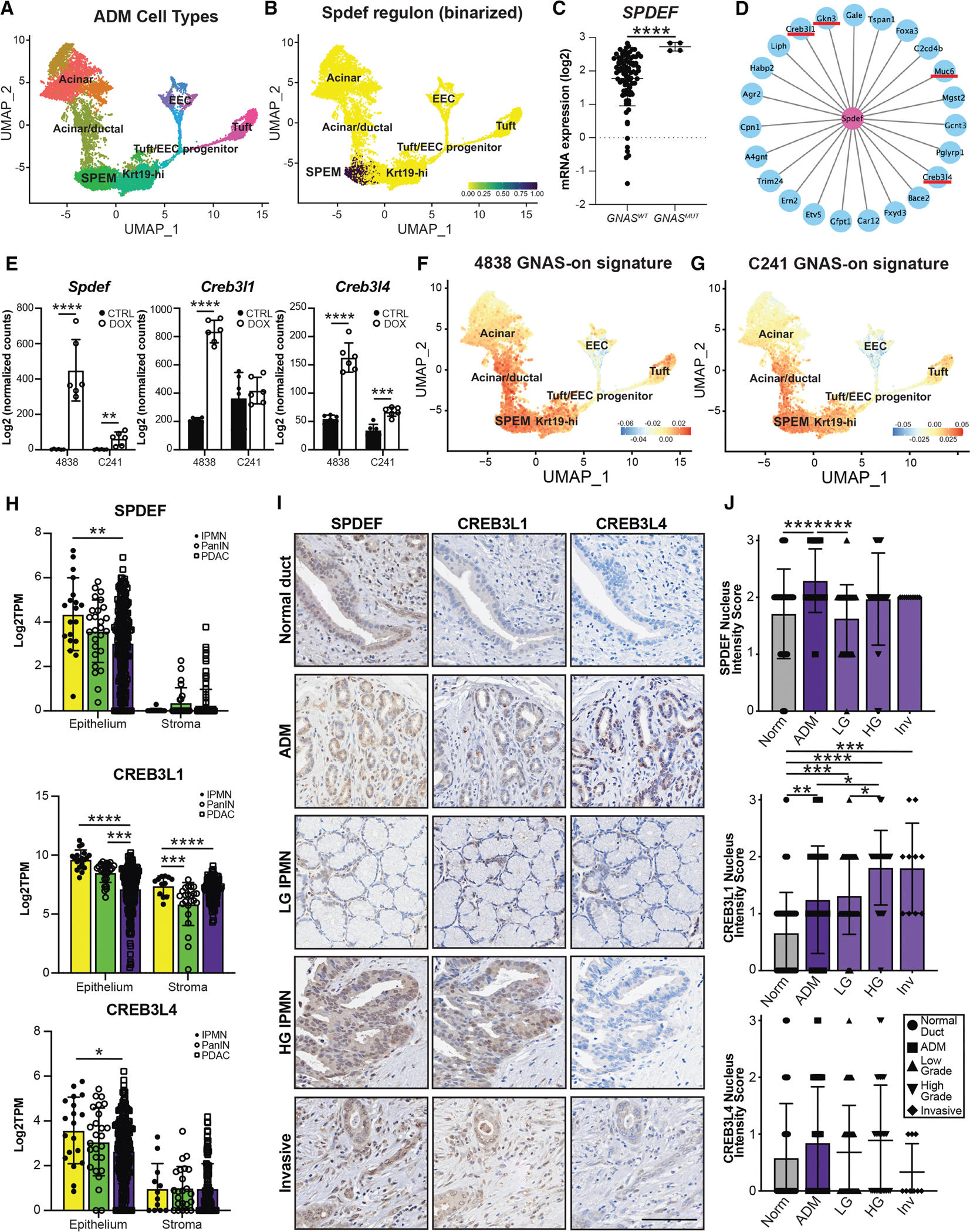
Identification of master regulator transcription factors of pyloric metaplasia (A) UMAP of scRNA-seq data from a murine model of pancreatitis showing the formation of a gastric SPEM.^13^ (B) Binarized activity of *Spdef* predicted by Regulon analysis overlaid on the UMAP from (A). (C) *SPDEF* expression in human PDAC tumors from cBioPortal with wild-type (*n* = 89/92) or mutant GNAS^R201C/H^ (*n* = 4/4). (D) Plot of *Spdef* targets predicted by Regulon analysis identifying gastric SPEM markers (*Gkn3* and *Muc6*) and transcription factors *Creb3l1* and *Creb3l4*. (E) Bar plots showing increased expression of *Spdef*, *Creb3l1*, and *Creb3l4*, determined by RNA-seq, in IPMN cell lines with DOX treatment and GNAS^R201C^ expression. (F and G) (F) DOX/GNAS-on gene signatures from IPMN cell line 4838 or (G) C241 overlaid on the UMAP in (A) showing overlap with predicted *Spdef* activity. (H) Bar plots comparing the expression (Log2TPM) of microdissected epithelium and stroma from Maurer et al.^19,20^ from IPMNs, PanIN, and PDAC for *SPDEF*, *CREB3L1*, and *CREB3L4*. (I-and J) Representative IHC and quantification for SPDEF, CREB3L1, and CREB3L4 conducted on 23 IPMN patient specimens, including normal ducts (Norm; *n* = 95–109), acinar-to-ductal metaplasia (ADM; *n* = 109), low-grade IPMN (LG; *n* = 110), high-grade IPMN (HG; *n* = 70), and invasive IPMN (Inv; *n* = 22). Scale bar, 100 μm. *p < 0.05; **p < 0.01; ***p < 0.005; and ****p < 0.001.

Previously, we showed that pancreatitis is characterized by a cell population resembling SPEM.^13^ To identify master regulator transcription factors driving this phenotype, we performed a PyScenic-based Regulon analysis on our pancreatitis scRNA-seq dataset (~13,000 cells), which identifies candidate factors by expression of known downstream target genes (Figure 3A). Among the candidate regulators, we identified the transcription factor *Spdef*, which has been shown to be the master regulator of mucin-producing goblet cell formation in the stomach, intestines, and lung (Figure 3B).^27–29^ Consistent with a role for driving mucin production, we found that *GNAS* mutations in human tumors are associated with both elevated mucin and *SPDEF* expression (Figures 3C and S5E) and that *GNAS*^*R201C*^ expression is sufficient to drive *Spdef* expression in *Kras*;*GNAS* cell lines (Figure 3E). Among the top 10% of predicted *Spdef* target genes, we identified several SPEM markers (*Gkn3* and *Muc6*), as well as additional transcription factors predicted to drive this phenotype (*Creb3l1* and *Creb3l4*; Figure 3D; Table S5). *GNAS*^*R201C*^ was sufficient to increase the expression of both *Creb3l1* (4838 cells) and *Creb3l4* (4838 and C241 cells; Figure 3E). Regulon analysis predicted that both *Creb3l1* and *Creb3l4* regulate the expression of canonical SPEM markers (*Aqp5*, *Tff2*, and *Muc6*, among others), as well as the expression of each other (*Spdef*, *Creb3l1*, and *Creb3l4*; Figures S6 and S7; Tables S6 and S7). To compare these data to *GNAS*^*R201C*^-induced changes in gene expression, we first overlaid the DOX-on gene signatures from both cell lines onto the pancreatitis scRNA-seq dataset and identified enrichment in the SPEM cluster (Figures 3F and 3G); expression patterns closely resembled those for *Spdef* (Figure 3B). We next examined changes in the expression of *Spdef*, *Creb3l1*, and *Creb3l4* target genes with DOX treatment and *GNAS*^*R201C*^ expression. We identified an increase in target genes in both cell lines for all three transcription factors, including SPEM markers (Figures S6–S9), suggesting that *GNAS*^*R201C*^ drives the expression of a pyloric metaplasia program through the activity of one or more of these transcription factors.

A recent study showed that the transcription factor NKX6–2 is expressed in the gastric subtype of IPMNs and that overexpression drives gastric marker expression in *KrasGNAS* cell lines.^30^ While we identified low *Nkx6–2* expression in the *in vivo* RNA-seq datasets interrogated in this study (Figure S10), it was not identified as a driver of SPEM in our Regulon analysis^13^ and was not expressed in *KrasGNAS* cell lines ± DOX (Files S2–3). Therefore, while NKX6–2 may drive the expression of gastric markers, our data suggests that it is not the mechanism by which *GNAS*^*R201C*^ does so.

To investigate the relevance of SPDEF, CREB3L1, and CREB3L4 to human IPMN and PDAC, we next examined their expression in patient samples. First, we interrogated the bulk RNA-seq dataset generated by Maurer et al. of IPMNs, PanIN, and PDAC.^19,20^ Expression of all three transcription factors was elevated in the epithelium of all disease states, as well as elevated *CREB3L1* in the stroma (Figure 3H). To examine protein expression, we next conducted IHC on 23 tissue samples collected from patients with IPMN ± associated PDAC and scored expression. Regions of interest encompassed normal ducts (*n* = 43–45), ADM (*n* = 39–41), LG IPMN (*n* = 51–54), HG IPMN (*n* = 27–33), and Inv PDAC (*n* = 9–10). Low expression of all three transcription factors was detected in a portion of normal ducts, with expression increasing in ADM and reaching significance for SPDEF and CREB3L1 (Figures 3I and 3J). While CREB3L4 expression remained low throughout IPMN to PDAC progression, SPDEF remained high, though the expression did not significantly increase between LG IPMN and PDAC. CREB3L1, though, did significantly increase with disease progression from normal ducts through IPMNs to PDAC (Figures 3I and 3J). Collectively, these data suggest a possible role for SPDEF and CREB3L1 as regulators of a pyloric phenotype in GNAS-mutated IPMN.

### Mutant GNAS drives major shifts in glycan species abundance

Consistent with GNAS^R201C^ driving a pyloric phenotype strongly defined by changes in mucin production, we noted significant upregulation of N-acetyl-galactosaminyltransferase *B4galnt3*, which synthesizes LacdiNAc (LDN) glycans (Figure 2E).^31^ Glycans are carbohydrate moieties that modify cell-surface receptor function and are a predominant feature of mucins.^32^ Specific glycans (e.g., acidic Lewis epitopes like CA19–9 and 3′-sulfo-Le^A/C^) are used clinically as biomarkers to detect high-grade dysplasia and cancer in IPMNs and to monitor therapeutic response and recurrence in PDAC.^33,34^ Forced expression of these Lewis epitopes in murine models suggests that such glycans are pathogenic and promote the development of PDAC.^33^ This is in contrast to LDN glycans, which have not been associated with high-grade dysplasia and cancer.

**Figure 4.**
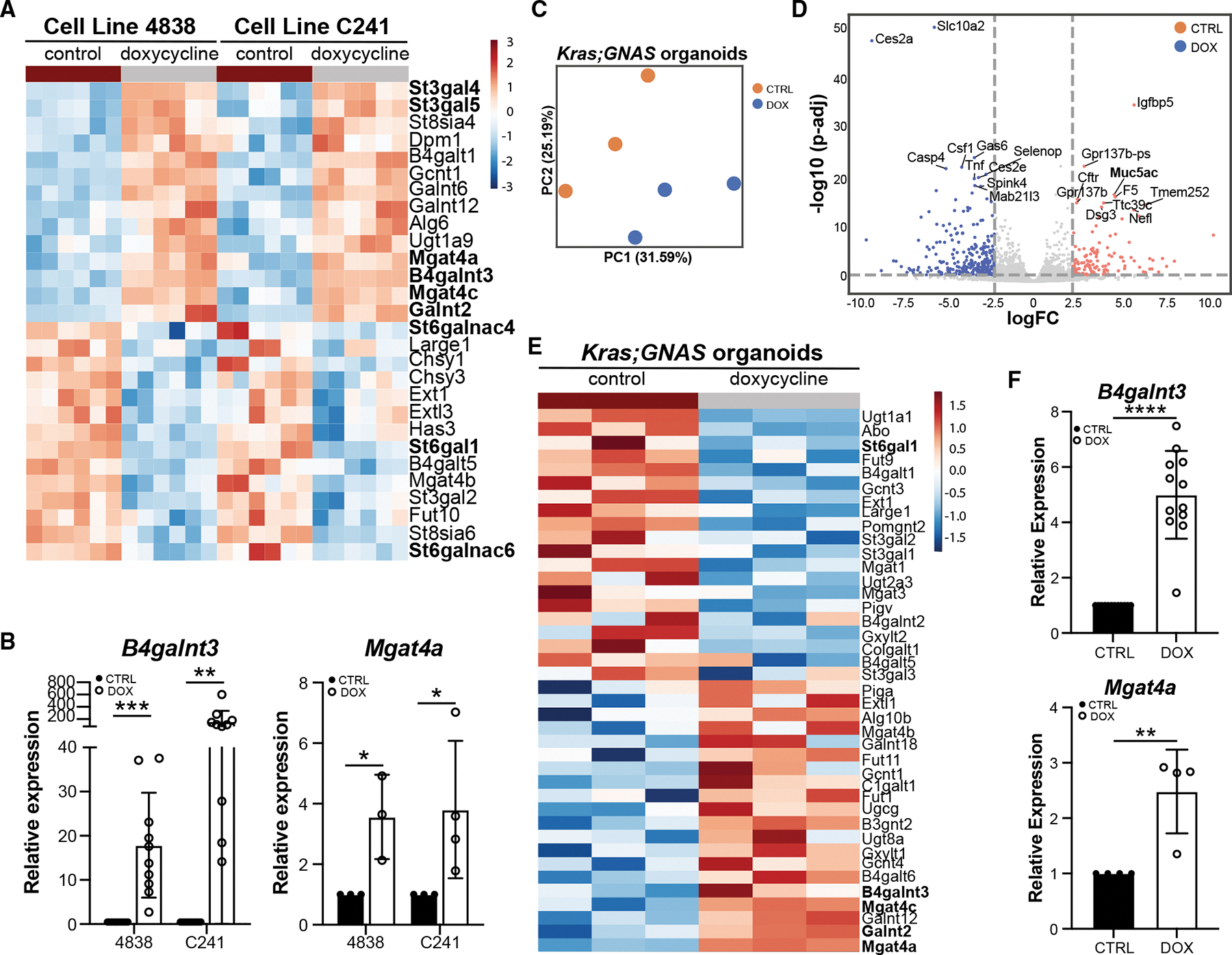
Mutant GNAS drives significant changes in glycosyltransferase gene expression (A) Heatmap of select glycosyltransferase gene expression in 4838 and C241 cells treated ± DOX. (B) RT-qPCR for either *B4galnt3* (*n* = 10) or *Mgat4a* (*n* = 3–4) in both cell lines ± DOX. (C) PCA of RNA-seq conducted on organoids generated from *Kras*;*GNAS* mice treated ± DOX (*n* = 3 biological replicates). (D) Volcano plot of differentially expressed genes in *Kras*;*GNAS* organoids ± DOX, highlighting the pyloric metaplasia marker *Muc5ac*. (E) Heatmap of select glycosyltransferase gene expression in *Kras*;*GNAS* organoids treated ± DOX. (F) RT-qPCR for either *B4galnt3* (*n* = 4 biological, 3 technical replicates) or *Mgat4a* (*n* = 4 biological replicates) in *Kras*;*GNAS* organoids ± DOX. *p < 0.05; **p < 0.01; ***p < 0.005; and ****p < 0.001.

To examine global changes in glycosyltransferase expression, we interrogated our RNA-seq dataset for the expression of 137 different enzymes (Figure S11A) and identified significant changes in several, including the upregulation of *Mgat4a*, which catalyzes the transfer of GlcNAc to generate branched glycan structures, consistent with an increase in mucin gene expression (Figures 4A and 4B). To determine if these changes are directly due to *GNAS*^*R201C*^ and are not a cancer-cell–specific effect, we generated organoids from murine *Kras*;*GNAS* pancreata lacking disease. Several lines (*n* = 3 mice) were propagated in culture, treated ± DOX to induce *GNAS*^*R201C*^, and underwent RNA-seq (Figure 4C; Table S8). Consistent with results in cell lines, we identified significant upregulation of *Muc5ac* (Figure 4D), in addition to glycosyltransferases *B4galnt3* and *Mgat4a* (Figures 4E and 4F) in DOX-treated organoids in contrast to controls. Similar gene expression changes, including pyloric markers *Aqp5* and *Creb3l1*, were identified in *GNAS* organoids ± DOX lacking *Kras*^*G12D*^ (Figures S11B–S11E). Collectively, these data show that *GNAS*^*R201C*^ alone or with *Kras*^*G12D*^ drives a pyloric phenotype, which includes significant changes in glycosyltransferase expression.

**Figure 5.**
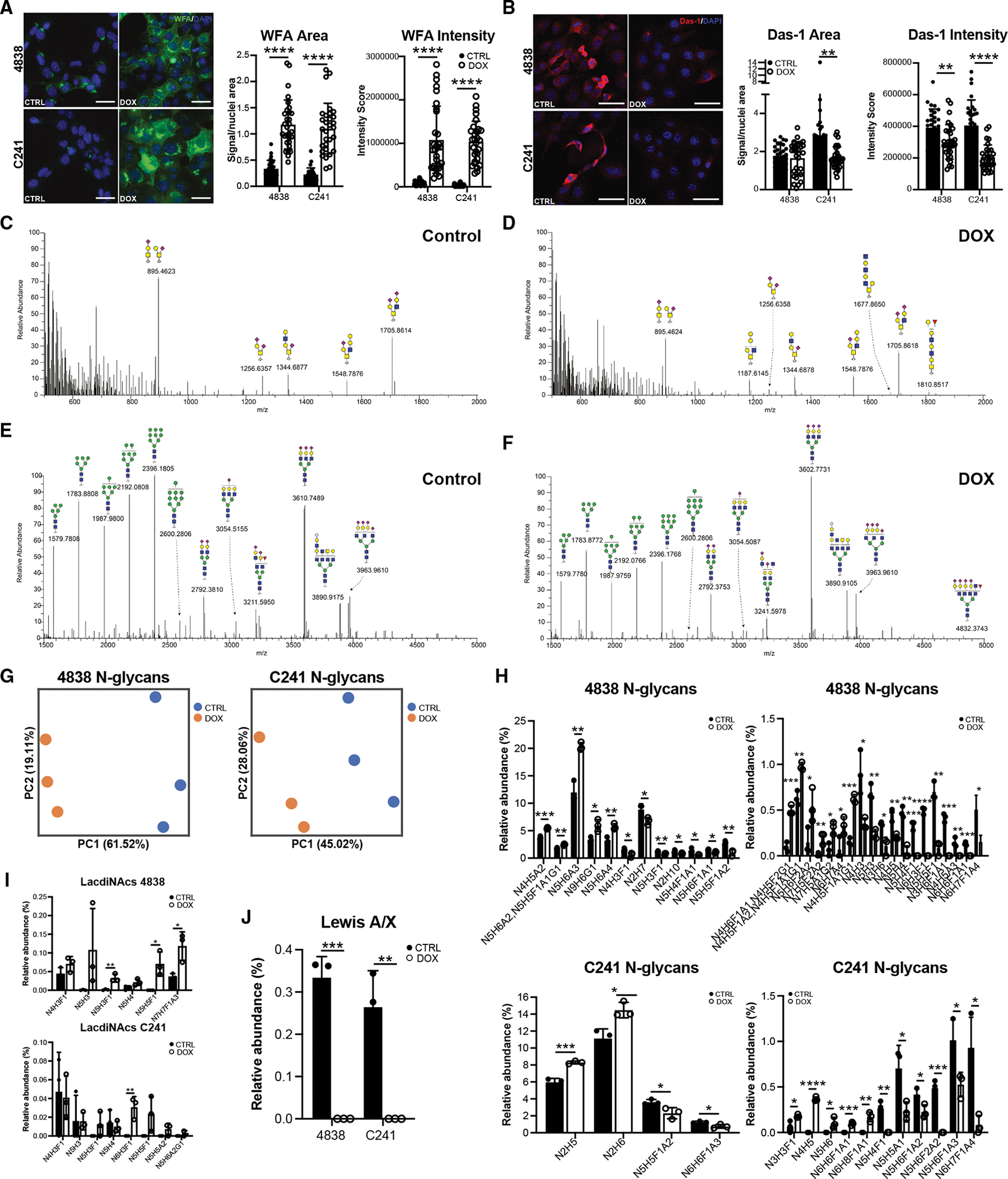
Mutant GNAS drives significant changes in N-glycosylation (A–F) (A and B) IF and quantification of (A) lectin WFA recognizing LDNs and (B) Das-1 antibody staining recognizing 3′-sulfo-Le^A/C^ in 4838 and C241 cells ± DOX (*n* = 3 biological replicates, 10 ROIs per sample). Scale bars, 50 μm. (C and D) Spectra of O-glycans in control (C) or DOX (D)-treated 4838 cells. (E and F) N-glycans identified by mass spectrometry in control (E) or DOX (F)-treated 4838 cells. (G) PCA of N-changes in 4838 or C241 cells ± DOX. (H–J) (H) Bar plots highlighting significant changes in N-glycans, (I) LDNs, or (J) Lewis^A/X^ antigen in both cell lines ± DOX (*n* = 3 biological replicates per cell line). *p < 0.05; **p < 0.01; ***p < 0.005; and ****p < 0.001.

To determine if changes in glycosyltransferase gene expression translate to changes in glycan abundance, both cell lines were treated ± DOX and then stained with lectins recognizing both terminal GalNAcs with a preference for LDNs^35^ (*B4galnt3*) and GlcNAcs (*Mgat4a* and/or *Gcnt2*, which bias toward GlcNAc high-branching glycans). As shown in Figure 5A, we saw a significant increase in both area and intensity of *Wisteria floribunda* (WFA) staining in both cell lines with DOX treatment, reflecting an increase in LDNs. Similarly, we saw an increase in GlcNAc modifications by lectin *Griffonia simplicifolia* (GSII) in both cell lines with *GNAS*^*R201C*^ (Figure S11F). Interestingly, we saw a significant decrease in the intensity of Das-1 staining with DOX, which recognizes 3′-sulfated Lewis A/C glycans (3′-sulfo-Le^A/C^), a marker for high-grade dysplasia and PDAC, consistent with *GNAS*^*R201C*^ driving a less aggressive phenotype (Figure 5B).^34,36^ To explore global changes in glycosylation induced by *GNAS*^*R201C*^, we performed glycosylation profiling by mass spectrometry (Figures 5C–5F; Table S9). While minor changes in O-glycan species were identified with DOX treatment (Figures 5C, 5D, and S12), we saw a significant shift in N-glycan abundance in both cell lines (Figures 5E–5H and S13). Among these changes, we identified a significant increase in LDN species, consistent with WFA lectin staining (Figure 5I). We also identified a loss of Lewis^A/X^ abundance in both cell lines (i.e., N5H4F1) with *GNAS*^*R201C*^ induction, consistent with a loss of Das-1 staining and a less-aggressive phenotype (Figure 5J). To confirm these changes *in vivo*, we performed imaging glycan mass spectrometry on pancreatic tissue sections from *Kras*;*GNAS* mice fed ± DOX chow for 22 weeks. While less sensitive than our cell-line analysis, we were able to identify a significant increase in glycan abundance in PanIN and IPMNs, as compared to acinar tissue, in fucosylation, sialylation, and predicted LDNs (Figure S14; Table S10). Altogether, these data demonstrate that *GNAS*^*R201C*^ drives the synthesis of LDNs, which is associated with a loss of Lewis epitopes.^31^ These changes in glycan species/abundance may represent a mechanism by which *GNAS*^*R201C*^ inhibits tumor aggressiveness.

### GNAS^R201C^-induced glycan changes drive an indolent phenotype

**Figure 6.**
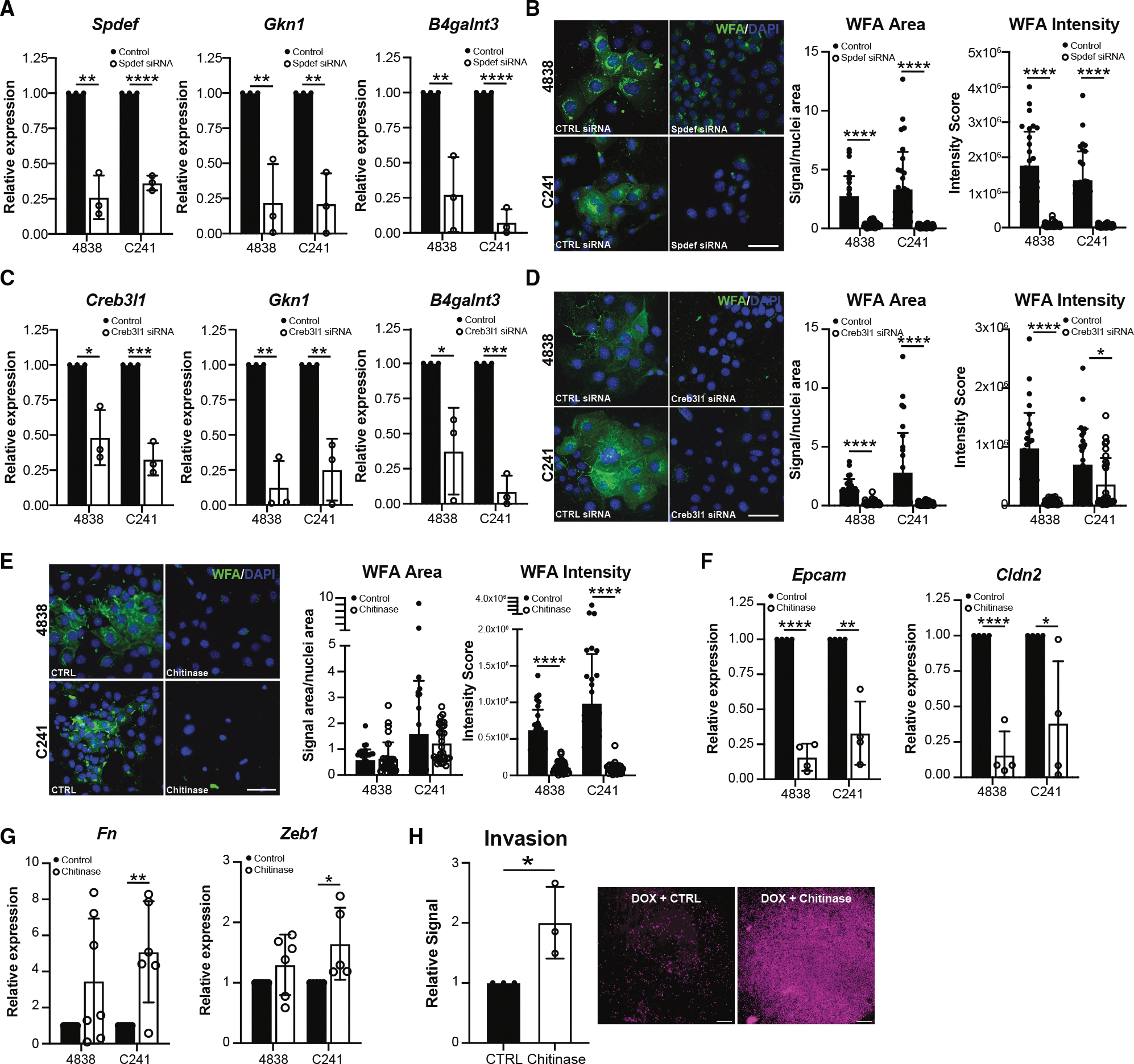
Mutant GNAS drives an indolent phenotype through LacdiNAc deposition (A) Bar plots of *Spdef*, *Gkn1*, or *B4galnt3* expression in 4838 or C241 cells treated with DOX and either control or *Spdef* siRNA, determined by RT-qPCR. (B) IF and quantification of lectin WFA in both cell lines treated with DOX and either control or *Spdef* siRNA. Scale bar, 50 μm. (C) Bar plots of *Spdef*, *Gkn1*, or *B4galnt3* expression in 4838 or C241 cells treated with DOX and either control or *Creb3l1* siRNA, determined by RT-qPCR. (D) IF and quantification of lectin WFA in both cell lines treated with DOX and either control or *Creb3l1* siRNA. Scale bar, 50 μm. (E) IF and quantification of lectin WFA in both cell lines treated with DOX and either control or chitinase. Scale bar, 50 μm. (Fand G) (F) Bar plots of *Epcam* and *Cldn2* or (G) *Fn* and *Zeb1* expression in 4838 or C241 cells treated with DOX and either control or chitinase, determined by RT-qPCR. (H) Quantification and representative images of invasion for C241 cells treated with DOX and either control or chitinase. Scale bar, 0.5 mm. *p < 0.05; **p < 0.01; ***p < 0.005; and ****p < 0.001.

Our Regulon analysis identified SPDEF and CREB3L1 as possible transcriptional regulators of the *GNAS*^*R201C*^-induced pyloric phenotype. To determine if either transcription factor is responsible for LDN deposition in response to *GNAS*^*R201C*^, we performed knockdown experiments using small interfering RNA (siRNA). In response to *Spdef* siRNA, we identified a significant decrease in the expression of *Spdef*, pyloric marker *Gkn1*, and *B4galnt3* in both cell lines by RT-qPCR (Figure 6A). We also saw a reduction in *GNAS* expression and in epithelial markers (*Epcam* and *Cldn2*; Figures S15A and S15B). Consistent with a loss of *B4galnt3*, we saw a significant decrease in the area and intensity of WFA lectin staining in both cell lines with *Spdef* siRNA as compared to control (Figures 6B, S15D, and S15E). In response to *Creb3l1* siRNA, we identified a significant decrease in the expression of *Creb3l1*, as well as *Gkn1* and *B4galnt3*, in both cell lines by RT-qPCR (Figure 6C). We again saw a decrease in *GNAS*, *Epcam*, and *Cldn2*, as well as a loss of WFA area and intensity with *Creb3l1* siRNA as compared to control (Figures 6C, 6D, S15F, and S15G). These data suggest that transcriptional regulators of the *GNAS*^*R201C*^ pyloric phenotype also regulate LDN abundance.

Previous studies have found that chitinase, an enzyme known to break down chitin, a non-mammalian structural protein found in fungal cell walls and the exoskeletons of crustaceans and insects, is also capable of hydrolyzing the GalNAcβ1–4GlcNAc bond found in several glycans, including LDNs.^37^ To determine if LDN cleavage impacts the *GNAS*^*R201C*^-induced phenotype, we treated DOX-incubated 4838 and C241 cells with chitinase. As shown in Figure 6E, we identified a significant decrease in WFA lectin intensity, consistent with LDN cleavage. Interestingly, we saw a significant decrease in epithelial markers *Epcam* and *Cldn2* in both cell lines with an increase in mesenchymal markers (*Zeb1* and *Fn1*) in C241 (Figures 6F and 6G). Consistent with a reversal of the *GNAS*^*R201C*^-induced MET phenotype, we saw an increase in cancer cell invasion with chitinase treatment in C241 cells (Figures 6H and S15H). Altogether, these data suggest that LDN deposition represents a mechanism by which *GNAS*^*R201C*^ inhibits cancer cell aggressiveness. Further studies are required to determine what cell-surface receptors are modified with LDNs and how this impacts their activity.

### Glycans may be used to distinguish low- from high-grade IPMNs

**Figure 7.**
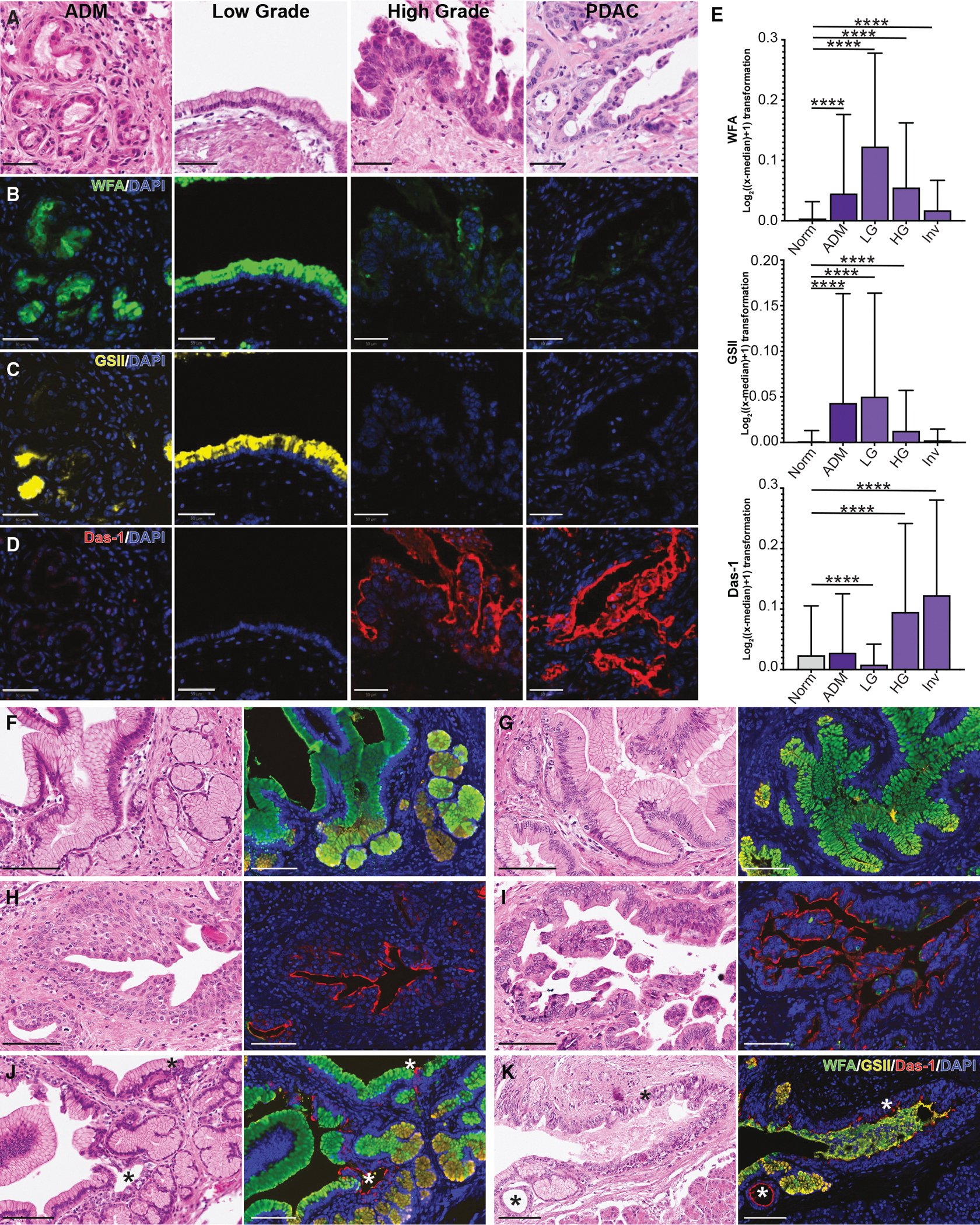
Glycans distinguish low- from high-grade IPMN and invasive disease (A) Representative H&E images of ADM, LG IPMN, HG IPMN, and invasive IPMN. Scale bars, 100 μm. (B–E) IF of (B) lectin WFA recognizing LDNs (green), (C) lectin GSII recognizing GlcNAcs (yellow), or (D) Das-1 recognizing 3′-sulfo-Le^A/C^ (red) in 27 IPMN patient samples and quantification (E). Scale bars, 100 μm. For WFA and GSII, *n* = 8,178 cells (Norm), 11,393 cells (ADM), 36,451 cells (LG), 19,277 cells (HG), and 22,423 cells (Inv). For Das-1, *n* = 3,724 cells (Norm), 11,806 cells (ADM), 24,800 cells (LG), 45,247 cells (HG), and 18,517 cells (Inv). Comparison to normal ducts shown. (F–K) Representative images of H&E and co-IF for WFA (green), GSII (yellow), and Das-1 (red) in low-grade IPMN (F and G), high-grade IPMN (H and I), and low-grade IPMN with foci of high-grade cells (J and K). Scale bars, 100 μm. ****p < 0.001.

In our analyses, we identified distinct glycosylation changes associated with low-grade disease and the *GNAS*^*R201C*^-driven, indolent phenotype (WFA+GSII+). To correlate these glycosylation findings with IPMN pathogenesis in patients, we selected ROIs of normal ducts, ADM, LG and HG IPMN, and Inv from H&E staining (27 patient samples; Figure 7A) and analyzed corresponding regions of IF for WFA, GSII, and Das-1 (3′-sulfo-Le^A/C^) abundance. Das-1 has been shown to bind high-grade and invasive PDAC and was found to be superior to imaging in risk-stratifying cystic lesions in the pancreas in a multicenter clinical trial.^34^ Consistent with our *in vitro* data, WFA and GSII were largely expressed in metaplasia and low-grade IPMNs (Figures 7B and 7C). As in prior reports, Das-1 was enriched in high-grade and invasive IPMNs (Figure 7D).^34^ We noted significant expression of glycans within the intraluminal mucin, correlating to tumor grade (Figures 7B–7K). Furthermore, combined staining of all markers highlighted small niches of morphologically high-grade cells within mostly low-grade IPMN (Figures 7J and 7K). Collectively, human glycan abundance largely recapitulated our experimental findings and may represent a new strategy to risk stratify IPMN patients.

## DISCUSSION

PanIN and IPMNs, the two most prevalent PDAC precursor lesions, are defined by mucin-producing cells harboring defining genetic alterations. The involvement of pyloric metaplasia has been described previously in murine pancreatitis and PanIN and now in human and murine IPMNs.^13,14^ Pyloric metaplasia-defining MUC5AC, CD44v9, and AQP5 are strongly expressed in ADM, and their co-expression is identified in dysplastic stages of IPMN progression (Figure 1). Furthermore, PanIN and IPMN pyloric metaplasia cells harbor similar whole-transcriptomic signatures, reciprocating gastric SPEM and foveolar pit cell lineages and raising the possibility of a conserved program between these organs.^13,14^ The identification of similar processes of injury and repair between gastrointestinal organs could lead to the discovery of targetable pathways for multiple inflammatory or pre-malignant conditions.

IPMNs differ clinically from PanIN through their capacity to be detected by routine imaging due to their larger size and cystic nature, filled with mucin. Some studies suggest that adenocarcinomas associated with IPMN have significantly better survival trends when compared to PanIN-associated PDAC, even with propensity-score matching.^38,39^ Consistent with this, we show here that GNAS^R201C^ drives a more indolent phenotype, suggesting that GNAS mutations may explain this disparity.^8^ While KRAS and GNAS drive pyloric metaplasia signatures, GNAS^R201C^ amplifies a mucinous phenotype, consistent with a role described for GNAS^R201H^.^13,40^ A recent clinical study showed that mucinous tumors from multiple tissue types are enriched for GNAS variants, suggesting a conserved role for GNAS mutations in tumorigenesis.^41^

Through Regulon analyses and siRNA-mediated knockdown, we found that mutant GNAS likely drives these changes through an SPDEF-CREB3L1 axis. Indeed, these markers increase in expression as early as the ADM phase, and CREB3L1 expression continues to increase with disease progression. While the transcription factor NKX6–2 has been shown to drive the expression of gastric markers in IPMNs,^30^ we did not identify an increase in expression in response to GNAS^R201C^, demonstrating that it is not likely to be the mechanism by which *GNAS*^*R201C*^ drives a pyloric phenotype in our model. As chronic injury, *Kras*^*G12D*^ and *NKX6–2* overexpression each independently drive the expression of components of pyloric metaplasia, we propose that this is a universal response to injury or mutation in the pancreas, with GNAS^R201C^ amplifying this phenotype.

SPDEF activity is a conserved process identified in the gastric, intestinal, and pulmonary epithelial response to injury and dysplasia and was recently shown to drive a mucinous phenotype in PanIN.^27–29,42^ However, a functional role for SPDEF in IPMNs had yet to be determined. Here, we identified a role for SPDEF in IPMNs in driving the expression of both pyloric markers and glycans. This further corroborates that not only is pyloric metaplasia conserved between organs but also distinct master regulators underlie these processes and the transition between cell states. While some studies have examined the functional role of individual pyloric markers in disease progression,^43^ it remains to be determined if SPEM and the subsequent addition of a foveolar pit lineage, or pyloric metaplasia, is protective or reflects a detectable sign of disease progression. Future studies assaying for this program may be useful in staging IPMNs, while functional studies on the overall role of this program may determine any benefit of inducing this differentiation program in advanced disease.

In addition to driving mucus cell formation through the SPDEF-CREB3L1 axis, we found that this axis drives major shifts in N-glycan species abundance (Figures 5 and 6). While other studies have reported changes in glycosylation in IPMNs,^44–54^ our work highlights a novel role for GNAS^R201C^ in driving these changes, including a significant increase in LDNs and GlcNAcs with a loss of the oncofetal epitope 3′-sulfo-Le^A/C^. Conserved changes in glycosylation underlie many processes throughout cancer progression and can drive changes in cell phenotype through regulation of cell-surface receptors.^55^ Receptors that stimulate cell proliferation, growth, and oncogenesis have been shown to have more canonical N-glycosylation motif sites (N-X-S/T), longer extracellular domains, and an increased number of sites per 100 amino acids than other classes of receptor (e.g., those involved in vascular formation and organogenesis). N-glycan branching and multiplicity are highly dependent on metabolic flux via the hexosamine pathway.^56^ A recent study demonstrated that induction of GNAS^R201C^ increases glycolysis in IPMN cell lines.^57^ Modulating glucose metabolism may represent a mechanism by which mutant GNAS modulates N-glycan composition.

N-glycosylation has been shown to differentially regulate EGFR/PI3K/ERK and TGF-β signaling, integrin-matrix interactions, pathways involved in cell proliferation, and EMT.^56,58^ Consistent with previous reports, we show here that mutant GNAS^R201C^ induces pyloric-like differentiation, inhibits cancer cell invasion, and drives a MET-like phenotype.^9,57,59^ Concomitant with MET, we have identified significant shifts in N-glycosylation, including a significant increase in LDN glycans. Chitinase treatment effectively removed LDN-containing glycans and was associated with a partial reversal of the MET phenotype. These results are consistent with a potential role for LDN in modifying cell-surface receptors involved in EMT and motility, although chitinase is not specific for LDNs and the underlying mechanisms require further investigation.^37^

We found that the formation of terminal LDNs correlates with a loss of 3′-sulfo-Le^A/C^. This may be a direct effect of LDN synthesis, as B4GALNT3 activity and the addition of N-acetyl-galactose in lieu of galactose prohibit the formation of Lewis epitopes.^31^ Here, we found that LDNs correlated with a benign phenotype and that LDN cleavage with chitinase promoted aggressive tumor behavior. Others have demonstrated that forced expression of Lewis epitopes in murine PDAC models led to greater inflammation as well as oncogenic transformation, suggesting that Lewis epitopes are carcinogens.^33^ Thus, it appears that mutant GNAS may induce an indolent phenotype in the KRAS background by (1) favoring the production of LDNs, which promote a benign phenotype, and (2) decreasing the expression of acidic Lewis epitopes that promote inflammation and cancer. As such, we propose that GNAS acts as a glycan rheostat controlling oncogenic potential. To our knowledge, this may be the first example of a genetic mutation that inhibits tumorigenesis by controlling glycan species abundance. While further studies are required to validate this proposed mechanism, we show here that LDN (WFA) and 3′-sulfo-Le^A/C^ (Das-1) staining are non-overlapping and specifically label low- and high-grade IPMN, respectively. The combined use of these biomarkers may ultimately aid in risk stratification of IPMN patients for surgery; however, validation is needed to determine their clinical utility. If confirmed, these markers could help prioritize patients who may benefit most from intervention while avoiding unnecessary surgery in others.

### Limitations of the study

Altogether, this study provides insight into the distinct clinical behavior, cystic form, and mucin-rich nature of some IPMNs. Mutant GNAS drives an indolent, pyloric phenotype in IPMNs through an SPDEF-CREB3L1 axis with potentially anti-tumorigenic glycosylation changes. While use of the *Kras*;*GNAS* cell lines to study IPMNs has many benefits, there are also several limitations, including the lack of genetic diversity and stromal cell populations. The *Kras*;*GNAS* mouse model primarily reflects early stages of IPMN development, whereas the available cell lines represent later stages of disease, together offering complementary but distinct perspectives on disease initiation and progression. Further validation of these findings in patient-derived organoids will be important to extend their translational relevance. Likewise, the current lack of comprehensive single-cell datasets in IPMNs restricted our analyses, underscoring the need for future studies in this area. Despite these constraints, our study establishes a mechanistic framework for understanding how mutant GNAS drives a pyloric phenotype characterized by distinct glycans using orthogonal approaches with the models currently available. Future studies will evaluate cell-surface receptor modification by glycans, how this impacts receptor activity, and if glycosylation can serve as a therapeutic target in addition to a biomarker.

## RESOURCE AVAILABILITY

### Lead contact

Requests for further information should be directed to Kathleen DelGiorno, kathleen.delgiorno@vanderbilt.edu.

### Materials availability

No new materials were generated in this study.

### Data and code availability

- Sequencing data generated in this study are available in the Gene Expression Omnibus (GEO: GSE244280). Unprocessed data related to the figures in this manuscript are available from the lead contact upon request.
- This paper does not report any original code.
- Any additional information required to reanalyze the data reported in this paper is available from the lead contact upon request.

## STAR★METHODS

### EXPERIMENTAL MODEL AND STUDY PARTICIPANT DETAILS

#### Human samples

A cohort of 41 patients with intraductal papillary mucinous neoplasms (IPMNs) ranging from low-grade dysplasia, high-grade dysplasia, to invasive IPMN-derived PDAC were selected from Vanderbilt University Medical Center’s institutional cohort with institutional review board approval (#101066). These samples were unmatched and came from distinct patients. Samples were graded by a board-certified gastrointestinal pathologist. Normal stomach body, small intestine, and colon tissues were used as controls to threshold signal intensity.

#### Mice

Mice were housed in accordance with National Institutes of Health guidelines in American Association for Accreditation of Laboratory Animal Care-accredited facilities at Vanderbilt University or M.D. Anderson Cancer Center. The Vanderbilt or M.D. Anderson Institutional Animal Care and Use Committees (IACUC) approved all animal studies. *LSL-Kras*^*G12D*/+^, *Ptf1a*^*Cre*/+,^ *LSL-rtTA-TetO-GNAS*^*R201C*^ (*Kras*;*GNAS*) mice have previously been described.^6,8,22^ Mice were given a diet containing doxycycline (Tusculum Feed Center) beginning at 8 weeks of age for a period of either 10 or 20 weeks.

### Cell culture

#### Culturing conditions and human GNAS^R201C^ induction

Murine cell lines generated from *Kras*;*GNAS* mice^8,22^ (4838 and C241) were cultured in RPMI with L-glutamine, 10% Tet system approved FBS, and 1 × antibiotic/antimitotic at 37°C, 5% CO_2_. To induce human *GNAS*^*R201C*^ expression, cells were stimulated with 1 μg/mL doxycycline for 48–72 h.

#### siRNA treatment

*Spdef* or *Creb3l1* knockdown was accomplished by siRNA mediated knockdown. siRNA for *Spdef*, *Creb3l1*, or a nonspecific control were transfected using lipofectamine RNAiMAX. Cells were seeded in 6 cm dishes with 1 μg/mL of doxycycline for 48 h. At 40% confluency, cells were transfected for a final concentration of 20 pmol. After 72 h, RNA was extracted, or cells were prepared for immunofluorescence.

#### Chitinase treatment

Chitinase treatment was performed in a similar matter with sells being seeded in 6-well plates with 1 μg/mL of doxycycline for 48 h. At 60% confluency, cells were treated with chitinase from *streptomyces griseus*. After 48 h, RNA was extracted, and cells were prepared for immunofluorescence.

### Pancreatic organoid isolation and culture

Organoids were established from the pancreata of 4, 6-week-old *Kras*;*GNAS* mice and 2, 6-month-old *GNAS* mice fed a normal chow diet. Briefly, pancreata were dissected, minced in Hank’s Balanced Salt Solution (HBSS) and incubated in digestion media containing 1 mg/mL collagenase IV, 10 μg/mL of DNAse I, 0.5 mg/mL of soybean trypsin inhibitor, and 5 mLs of dispase diluted in HBSS. Tissues were digested for 8–10 cycles by incubating in digestion media with gentle shaking at 37°C for 10–20 min per cycle. At the end of each cycle, cells were collected, the digestion buffer was neutralized with wash buffer (10% FBS in HBSS) and cells were placed on ice. After the last digestion, dispersed cells were filtered through a 70 μm strainer and pelleted at 1,000 × g for 5 min, embedded in Matrigel and organoid media was added. Pancreatic organoid media consisted of Advanced DMEM/F12 supplemented with 10% Noggin conditioned media, 10% R-spondin conditioned media, 1× B27, 10 mM nicotinamide, 1.25 mM N-acetylcysteine, 50 ng/mL of mEGF, 100 ng/mL of mFGF-10, 10 nM hGastrin I, and 500 nM of A83–01. Y-27632 was added to media directly after isolation but removed in following passages. Organoids were maintained in 5% CO_2_ at 37°C and passaged every 4–7 days. To induce GNAS^R201C^ expression, organoids were treated with 2 μg/mL doxycycyline for 72 h and RNA was extracted following manufacturer’s protocol (Zymo).

## METHOD DETAILS

### Immunoblotting

For analysis of GNAS^R201C^ protein expression, immunoblotting was performed. Briefly, cells were lysed with RIPA buffer with 1% protease/phosphatase inhibitors, incubated on ice for 10–30 min and centrifuged at 10,0000 × g for 10 min at 4°C. Protein concentration was estimated using a Qubit Protein Assay Kit per manufacturer’s instructions. Lysates were diluted in 4× Laemelli buffer with 1:10 beta-mercaptoethanol, boiled at 95°C for 5 min, and ran on Bio-Rad Mini-Protean 4–20% TGX gels. Proteins were transferred to a PVDF membrane using the Bio-Rad Trans Blot Turbo System. Blocking was done in 5% non-fat milk diluted in Tris-buffered saline with 0.1% Tween 20 (TBS-T) for 1 h at room temperature (RT). The primary antibodies (Table S1) were diluted in 5% non-fat milk and incubated in a rocker at 4°C overnight. The membrane was washed 5 times in TBS-T and incubated in mouse anti-rabbit HRP secondary (Santa Cruz, 1:10,000) diluted in 5% non-fat milk for 1 h at RT. The membrane was then washed 5 times in TBS-T and imaged with Immobilon HRP Substrate and using an Amersham AI600 imaging system.

### RNA extraction and RT-qPCR

For quantitative reverse-transcriptase PCR (RT-qPCR), RNA was isolated using Quick-RNA MiniPrep kits per manufacturer’s protocol, and concentration was measured using NanoDrop 2000. RT-qPCR was performed using a Luna Universal One-Step RT-qPCR Kit according to manufacturer’s protocol and analyzed using Bio-Rad CFX96 system and CFX Manager 3.1. Expression was calculated on CFX Manager 3.1 using *Rplp0* as a reference gene relative to the – DOX controls. Primers are listed in Table S2.

### Immunocytochemistry

For immunofluorescence, cells were seeded on a Matrigel-coated coverslip (1:50 dilution) and fixed with 4% paraformaldehyde (PFA) for 15 min at room temperature. Following fixation, cells were washed 5 times in phosphate buffered saline (PBS) and blocked and permeabilized using buffer containing 1% BSA (*w/v*), 5% donkey serum (*v/v*), 5% goat serum (*v/v*) and 0.3% Triton X- for 30 min at room temperature. Coverslips were washed in PBS 5 times. For primary incubation, primary antibodies (Table S1) were diluted in blocking buffer (5% donkey serum, 1% BSA and 0.05% Triton X-) and incubated at 37°C for 2 h. Coverslips were washed 5 times in PBS and secondary antibody diluted in blocking buffer was added. Secondary antibodies were incubated at 37°C for 1 h. Coverslips were washed 5 times in PBS, mounted with ProLong Gold Antifade Mountant with DAPI and imaged with a 20× objective on an Olympus VS200 Slide Scanner (Olympus, Tokyo, Japan).

### Invasion assays

Invasion assays were performed using Corning BioCoat Matrigel Invasion Chambers with 8.0 μm PET Membrane. Assays were prepared according to manufacturer protocol, and 125,000 cells were plated per well. After 24 h, cells on the membrane were fixed with 4% PFA for 15 min and stained with 0.2% *w/v* crystal violet for 15 min and washed in distilled water. Membranes were carefully cut out of the inserts, mounted, and imaged with a 20× objective on an Olympus VS200 Slide Scanner.

### Multiplex immunohistochemistry (MxIHC)

Formalin-fixed paraffin-embedded tissues from the patient cohort were sectioned at 4 μm, heated to 60°C for 30 min, deparaffinized in xylenes, and rehydrated in an ethanol gradient. Slides were stained with Mayer hematoxylin, coverslipped with 30% glycerol in PBS and scanned with an Olympus VS200 slide scanner. Slides were de-coverslipped by immersion in PBS and underwent antigen retrieval in 10 mM sodium citrate pH 6.0 with a microwave set at maximum power until boiling bubbles appeared, reduced to minimum power for 20 min, and left at RT for 30 min. Slides were treated with 3% hydrogen peroxide for 10 min, blocked with Protein Block, Serum-Free (Dako) for 10 min, and incubated with the primary antibody overnight. Secondary antibodies were incubated for 1 h, and the signal was revealed with AEC+ High Sensitivity Substrate Chromogen. The slides were then coverslipped with 30% glycerol in PBS. Slides were scanned with the Olympus VS200. Slides were de-coverslipped by immersion in PBS and underwent a 2-min double-distilled water, 2 min 70% ethanol, 2 min 95% ethanol, 2 min ethanol, 2-min double-distilled water sequence to eliminate the 3-amino-9-ethylcarbazole (AEC) chromogen. Slides then restarted the previous sequence at the antigen retrieval step until all primary antibodies (Table S1) were performed. Scans were loaded in QuPath v0.4.0^21^ and registered with the image-combiner v0.3.0 package.

### Standard histological staining

Murine and patient tissues were cut in 5 μm or 4 μm sections, respectively, mounted, and stained as previously described.^13^ Sections were deparaffinized in xylenes, rehydrated in a series of graded ethanols, and then washed in PBS-T and PBS. Endogenous peroxidase activity was blocked with a 1:50 solution of 30% H_2_O_2_:PBS followed by microwave antigen retrieval in 100 mM sodium citrate, pH 6.0. Sections were blocked with 1% bovine serum albumin and 5% normal goat serum in 10 mM Tris (pH 7.4), 100 mM MgCl_2_, and 0.5% Tween 20 for 1hr at room temperature. Primary antibodies (Table S1) were diluted in blocking solution and were incubated on tissue sections overnight. Slides were then washed, incubated in streptavidin-conjugated secondaries (Abcam), and developed with DAB substrate (Vector). Periodic Acid Schiff/Alcian blue (PAS/AB) staining was performed per the manufacturer’s instructions. For standard IHC on human tissue sections, antigen retrieval was performed with pH 6.0 citrate buffer in a pressure cooker at 105°C for 15 min, with a 10-min cool down. Blocking was performed in 0.03% H_2_O_2_ containing sodium azide for 5 min and primary antibodies (Table S1) were incubated for 60 min before detection (Dako EnVision+ System-HRP labeled Polymer) for 30 min and development for 5 min. All slides were scanned on the Olympus VS200.

### Bulk RNA-sequencing

RNA was extracted from either 4838 or C241 cells (+/− doxycycline) or organoid lines using Quick-RNA MiniPrep and quality was measured using an Agilent Bioanalyzer; RNA Qubit assay was performed to measure RNA quantity. Poly(A) RNA enrichment was conducted using NEBNext Poly(A) mRNA Magnetic Isolation Module (NEB E7490), and the sequencing library was constructed using the NEBNext Ultra II RNA Library Prep Kit (E7765L) following the manufacturer’s instructions. End repair, A-tailing, and adapter ligation was performed to generate the final cDNA library. Library quality was assessed using a Bioanalyzer and quantified using a qPCR-based method with the KAPA Library Quantification Kit (KK4873) and the QuantStudio 12K instrument. 150 bp paired-end sequencing was performed on the NovaSeq 6000 platform targeting 50M reads per sample.

### Glycosylation profiling of cell lines

Cells were lysed using 50 mM ammonium bicarbonate buffer, treated with dithiothreitol (DTT), and then incubated at 50°C for 30 min. The samples were then desalted using a 3 kDa molecular weight cutoff (MWCO) filter with 50 mM ammonium bicarbonate buffer and ultrasonicated. A BCA assay was used to quantify protein, and an equal amount of protein was further processed for N-glycan release. Samples were then treated with PNGaseF at 37°C for 48 h and released N-glycans were collected using a 10kDa cutoff spin filter followed by lyophilization. O-glycoproteins were retrieved from the top of the spin filter and lyophilized before proceeding to β-elimination. Both released N- and O-glycans were permethylated using methyl iodide in the presence of NaOH-DMSO, as previously described.^75^ Permethylated glycans were analyzed using direct injection electrospray ionization-mass spectrometry (ESI-MS) on an Orbitrap Eclipse Tribrid mass spectrometer. Glycan abundance (%) was calculated separately for each sample based on peak intensity. The N-glycan and O-glycan structures were assigned based on precursor masses (Sodium Adducts), MS/MS fragment ions, and knowledge of the mammalian biosynthetic pathway. Proposed structures do not reflect linkage analysis.

### Glycan imaging mass spectrometry

FFPE tissue sections were prepped as previously described.^76^ Briefly, slides were incubated for one hour at 60°C followed by deparaffinization using a wash series consisting of: xylenes for 3 min, xylenes for 3 min, 100% ethanol for 1 min, Carnoy’s solution (60% ethanol, 30% chloroform and 10% glacial acetic acid) for 3 min, Carnoy’s solution for 3 min, Carnoy’s solution for 3 min, 100% ethanol for 1 min, 95% ethanol for 1 min, 70% ethanol for 1 min, 150 mM ammonium formate for 1 min, 150 mM ammonium formate for 1 min. Samples were placed in a vacuum desiccator for 10 min. Autofluorescence microscopy was collected from each slide using a Zeiss AxioScan Z1 slide scanner with a 10× objective using the DAPI (excitation wavelength 353 nm), GFP (excitation wavelength 488 nm), and dsRed (excitation wavelength 572 nm) filter sets using exposure times of 20 ms, 60 ms, and 250 ms, respectively. Thermal denaturation was performed in 10 mM citraconic anhydride for 30 min in a preheated steamer followed by buffer exchange with milli-q water. Samples were placed in a vacuum desiccator for 10 min. Samples were then coated with 0.1 μg/μL PNGase F Prime in HPLC-grade water using an HTX M5 robotic sprayer (HTX Imaging, Chapel Hill, NC) using the following conditions: 37°C nozzle temperature, ambient stage temperature, 10 psi-compressed air, 15 passes, 1200 mm/min velocity, 3 mm offset, CC spray pattern, 40 mm nozzle height, no dry time. A syringe pump equipped with a 500 μL Hamilton syringe with a 3.26 mm diameter was used for enzyme spraying with a 25 μL/min flow rate. Enzymatic digestion was performed in a preheated digestion chamber containing 5 mL milli-q water at 38°C for 2 h α-Cyano-4-hydroxycinnamic acid matrix solution at a concentration of 7 mg/mL in 50% acetonitrile containing 0.1% TFA was applied to the slides using an HTX M5 sprayer with the following conditions: 72°C nozzle, 10 psi compressed air, ambient stage temperature, 0.1 mL/min flow rate, CC spray pattern, no dry time, 40 mm nozzle height, 10 passes, 1300 mm/min, 3 mm offset. All robotic spraying steps were performed at 76°F and 60% relative humidity. Matrix-assisted laser desorption/ionization (MALDI) imaging mass spectrometry (IMS) was performed using a Bruker timsTOF Flex in positive ionization mode with an m/z from 500 to 4000, 200 laser shots at a frequency of 10,000Hz with a laser fluence of 39% and a pitch of 20 μm. Ion images were visualized using Bruker SCiLS Lab.

## QUANTIFICATION AND STATISTICAL ANALYSIS

### Immunofluorescence analysis

Cell line IF images were analyzed for corrected total cell fluorescence (CTCF) using Fiji (National Institutes of Health).^77^ Briefly, representative regions of interest (ROIs) in each image were selected using the freehand selection tool in the experimental channel. The area, mean, integrated density, and raw integrated density were calculated for each ROI. The same values were also taken from the background. To calculate the CTCF, the following formula was used: raw integrated density – (area*average background mean). For the area analysis, images were split into separate channels, thresholded, and the area of both the DAPI and experimental channel were measured. To normalize for the number of cells in each ROI, the area of the antibody or lectin stain was normalized to the area of the nuclear stain.

### IHC quantification

#### MxIHC quantification

Areas measuring 250 × 250 μm^2^ were acquired blindly on the hematoxylin layer, querying normal ducts, acinar-to-ductal metaplasia (ADM), low-grade dysplasia (LG), high-grade dysplasia (HG), and invasive disease under the supervision of a board-certified pathologist (VQT). Up to 3 areas of normal, ADM and INV, and up to 5 areas of LG and HG, were selected per patient (when present). An automated watershed threshold pixel detection was adjusted based on the staining patterns on the control gastric, small intestinal, and colonic tissues. Automated AEC deconvolution was performed in QuPath v0.4.0^21^ by loading them as an H-DAB slide. An automatic calculation of the surface area with the AEC color deconvolution intensity over the threshold was then generated. Pseudocolors were given to each stain for the figures.

#### Monoplex quantification of SPDEF, CREB3L1, and CREB3L4

Scans of SPDEF, CREB3L1, and CREB3L4 were obtained with the VS200 and loaded in QuPath v0.4.3.^21^ Color deconvolution of the hematoxylin stain was performed to select up to 3 areas of normal ducts, ADM, LG, HG, and INV. By consensus, one student and a board-certified pathologist (VQT) scored the nucleus and cytoplasm of cells on a scale of 0–1-2–3. Cell type quantification in murine IPMN. Scans of H&E staining from *Kras*;*GNAS* mice ± doxycycline chow for 10 or 20 weeks (*n* = 3/condition, *n* = 12 total) were scored by a pathologist (VQT) and areas of IPMN and/or PanIN lesions were identified (*n* = 3, up to 6 regions/slide). Serial sections were stained for DCLK1 (tuft cells), synaptophysin (enteroendocrine cells), or PAS/AB (all mucins) and 1–6 lesions were identified per area of IPMN and/or PanIN (when present). All steps of analysis were performed blinded. Positive DCLK1 or synaptophysin cells were manually counted and divided by the number of nuclei per lesion to identify percent of that lesion constituted by a given cell type. To quantify PAS/AB mucin staining, lesions were annotated as regions of interest and manually drawn in FIJI.^77^ Total signal and PAS/AB signal were thresholded to include only stained areas and percent positive area was calculated.

### Analysis of published single-cell RNA sequencing datasets

Processed count matrices from Bernard et al.,^18^ were provided by the authors. Data analyses were executed using R (version 4.4.2, 2024–10-31 ucrt), tailored for a 64-bit windows platform. R integrated package utilized in the study consisted of ‘Seurat(‘5.3.0’)’,^78,79^ ‘ggplot2(‘3.5.2’)’, ‘SeuratObject(‘5.1.0’)’, ‘patchwork(‘1.3.1’)’, ‘harmony(‘1.2.3’)’, ‘dplyr(‘1.1.4’)’, ‘devtools(‘2.4.5’)’. Individual Seurat objects were established from the six patient datasets from Bernard et al. and merged into one cohesive Seurat object. Consolidated data was filtered to cells with a minimum of 300 genes, manifesting less than 15% mitochondrial genes. The unified dataset was normalized and scaled using NormalizeData() followed by ScaleData(). Post scaling, data was subjected to Principal Component Analysis (PCA) to demarcate inherent patterns. Exploiting the dimensionality reduced space (spanning the first 10 principal components), cellular neighborhoods were mapped with the ‘FindNeighbors()’ function (dims = 1:10,k.param = 10) and clusters were discerned using ‘FindClusters()’ with a resolution parameter set at 0.5. UMAPs labeled by sample or for individual gene markers (‘CD44′,’MUC2′,’TFF2′,’PTPRC’,’EPCAM’,’AQP5′,’MUC5AC’,etc.) were plotted for visualization using FeaturePlot() and DimPlot() functions.

### cBioPortal analysis

mRNA expression (log2) values for individual genes (*B4GALNT3*, *CREB3L1*, *CREB3L4*, *MUC5AC*, *MUC5B*, AND *SPDEF*) from pancreatic tumors were downloaded from Bailey et al.^80^ off cBioPortal^81–83^ as.txt files. Expression data was unified into one dataset using R (version 4.4.2, 2024–10-31 ucrt) tailored for a 64-bit windows platform. Using the t.test () function a two-tailed Welch two sample *t* test was run between mutant (*n* = 4) and non-mutant (*n* = 92) GNAS tumors for individual genes. Log2 expression was plotted in GraphPad Prism between non-mutant and mutant GNAS samples.

### Bulk human RNA- sequencing analysis

Compartment-specific gene expression profiles of human IPMN (*n* = 19), PanIN (*n* = 26) and PDAC (*n* = 197) epithelia or stroma were generated using laser capture microdissection with subsequent RNA sequencing as previously described.^19,20^ Stromal samples were matched and collected adjacent to the diseased tissue. IPMN samples were acquired from non-tumor pancreatic resections and the number of IPMN-associated PDAC was negligible. Differentially expressed genes between human IPMN and PanIN were identified leveraging a generalized linear model as implemented in DESeq2 R package^84^ and genes exhibiting a false discovery rate ≤0.05 were considered significantly differentially expressed.

### Bulk murine cell line RNA collection and sequencing analysis

Raw sequencing data (FASTQ files) obtained from the NovaSeq 6000 was subjected to quality control analysis, including read quality assessment. Real Time Analysis Software (RTA) and NovaSeq Control Software (NCS) (1.8.0; Illumina) were used for base calling. MultiQC (v1.7; Illumina) was used for data quality assessments. Paired-end RNA sequencing reads (150bp long) were trimmed and filtered for quality using Trimgalore v0.6.7 (https://doi.org/10.5281/zenodo.7598955). Trimmed reads were aligned and counted using Spliced Transcripts Alignment to a Reference (STAR) v2.7.9a with the -quantMode GeneCounts parameter against the mm39 mouse genome and GENCODE comprehensive gene annotations (Relsease M31).^68^ ~50–100 million uniquely mapped reads were acquired per sample. Sample read counts were normalized and differential expression was performed using DESeq2 v1.34.0.^84^ Genomic features counted fewer than five times across at least three samples were removed. False discovery rate adjusted for multiple hypothesis testing with Benjamini-Hochberg (BH) procedure *p* value <0.05 and log2 fold change >1 was used to define differentially expressed genes. Gene set enrichment analysis (GSEA) was performed using the R package Clusterprofiler^85^ with gene sets from the MSigDB database.^86^

### Organoid bulk RNA-seq analysis

#### Differential gene expression analysis and visualization

RNA-seq raw count data was used as input to the DESeq2(1.44.0) R package DESeq() function. Differential gene expression results were computed, comparing the control and doxycycline treated groups. Variance stabilizing transformation was performed using the DESeq2 varianceStabilizingTransformation() function on the RNA-seq raw count data, and the resulting transformed data (vsd data) was used for down-stream analysis. Differential gene expression results output from DESeq2 were visualized via volcano plots. Matplotlib(3.7.5)’s axes.Axes.scatter() function was used to plot −log10 transformed adjusted *p*-values over log2 fold change values for each gene. Genes with absolute log2 fold change values ≥ 2 and adjusted *p*-value ≤ 0.05 were highlighted.

#### Heatmap visualization

Scanpy(1.9.3) function^69^ scanpy.pl.heatmap() was used to generate the heatmaps. Vsd count matrices output from DESeq2 were used as input to the heatmap function. Heatmap colors represented the gene expression values that were normalized by library size, log1p transformed and *Z* score scaled. Genes were ordered by ascending differential gene analysis test-statistics on the heatmap.

#### Principal component analysis (PCA) and visualization

For *Kras*;*GNAS* and GNAS organoid gene expression, the DESeq vsd count matrices were used to compute the principal components (PC) of the data. For cell line glycan abundance analysis, the mass spectrometry glycosylation profiling data were used. Data matrices were normalized by library size, arcsinh-transformed and *Z* score scaled for the PCA analysis. scanpy.tl.pca() function was used to perform the PCA, with transformed matrices and other default arguments of the function as inputs. Scatterplots of the first two PC values were generated using the scanpy.pl.pca() function, with data points colored by conditions of interest.

### pySCENIC and gene regulatory network (GRN) inference

To infer the GRN in pyloric metaplasia of the pancreas, we performed SCENIC using pySCENIC functions on a scRNA-seq dataset of murine pancreatitis.^13,74^ This protocol allows the reconstruction of regulons (TF and known target genes) from gene co-expression data, assesses regulon activity in single cells, and can be used to find regulon-enriched cellular clusters. Specifically, we ran v0.11.0 of pySCENIC in a Singularity container built from the Docker Hub image, on ACCRE, Vanderbilt’s High Performance Computing cluster. Following the quality control and feature selection performed in Seurat, we exported its raw counts to a matrix that was then converted to a LOOM file. Alongside a list of 1170 mouse TFs, this gene expression matrix served as input for calculating gene co-expression modules via GRNBoost2. To account for the stochastic nature of GRNBoost2, we calculated the co-expression modules 100 times and then retained only TF/target gene associations that exist in at least 80% of the runs. We then merged the results of these 100 runs as a left outer join operation and averaged the IM values reported for each association. This consensus GRN was then used as input for module pruning, where we filtered out indirect gene targets lacking the *cis*-regulatory motif associated with the TF. This step used SCENIC’s RcisTarget and ranking databases for motifs (mm9-tss-centered-5kb-7species.mc9nr. feather) in the promoter of the genes [up to 500 base pairs (bp) around the transcriptional start site (TSS)]. The resulting co-expressed TF-target genes are then grouped into regulons. Lastly, the activity of the regulons was computed using SCENIC’s AUCell function, which uses the “area under the curve” (AUC) to calculate whether a subset of the input gene set is enriched within the expressed genes for each cell. These activity data were further binarized (assigned an ON or OFF value, per regulon, per cell) by threshold on the AUC values of the given regulon. Both the AUCell and binarized regulon activity matrices were integrated into Seurat object via the “CreateAssayObject” function, for downstream analysis and visualization.^87^ UMAP visuals of the binary and AUC metrics were created from Seurat’s DimPlot() function. Heatmap visuals of binary regulon matrix were performed by ComplexHeatmap R package.^88^ Network plots were created from Cytoscape from top 10% based on IM reported for TF/target association from the coexpression modules.^89^

### Statistical analysis

Statistical analyses and data processing were performed in ImageJ or Prism (GraphPad, San Diego, CA). Statistical significance was calculated by either 2-tailed unpaired t-tests assuming equal variance or 1-way analysis of variance as indicated in the figure legends. The number of samples (n) is also noted in the figure legends. Data are expressed as mean ± standard deviation. Figures were generated with Adobe Photoshop and Illustrator.

## Supplementary Material

1

2

4

5

6

7

8

9

10

11

SUPPLEMENTAL INFORMATION

Supplemental information can be found online at https://doi.org/10.1016/j.celrep.2025.116684.

## Figures and Tables

**KEY RESOURCES TABLE:** 

REAGENT or RESOURCE	SOURCE	IDENTIFIER

Antibodies		

Mouse anti-rabbit IgG-HRP	Santa-Cruz	Cat# sc-2357; RRID: AB_628497
Rabbit polyclonal anti-AQP5	Sigma-Aldrich	Cat# HPA065008; RRID: AB_2685401
Rabbit monoclonal anti-CD44 v9 (Clone RV3)	Cosmo Bio	Cat# CAC-LKG-M003; RRID: AB_3101985
Rabbit monoclonal anti-CD44 v10-e16 (Clone RV3)	Cosmo Bio	Cat# CAC-LKG-M002; RRID: AB_2910608
Rabbit polyclonal anti-CREB3L1	Sigma-Aldrich	Cat# HPA024069; RRID: AB_1854750
Rabbit polyclonal anti-CREB3L4	Sigma-Aldrich	Cat# HPA038122, RRID: AB_10669516
Mouse monoclonal anti-Das-1 antigen (3' Sulfo-Lewis A/C)	Jeff Brown Lab	N/A
Rabbit polyclonal anti-DCAMKL1	Abcam	Cat# ab37994; RRID: AB_873538
Rabbit monoclonal anti-GAPDH (D16H11)	Cell Signaling Technologies	Cat# 5174; RRID: AB_10622025
Rabbit polyclonal anti-GNAS ^R021C^	GeneTex	Cat# GTX135412; RRID: AB_2887494
Lectin GS-II, Alexa Fluor^™^ 488 Conjugate	Invitrogen	Cat# L21415
Mouse monoclonal anti-MUC5AC	Invitrogen	Cat# MA5-12178; RRID: AB_10978001
Rabbit polyclonal anti-SPDEF	Lifespan Bioscience	Cat# LS-C499857
Rabbit monoclonal anti_SYP	Cell Marque	Cat# 336R; RRID: AB_3096182
Lectin WFA, FITC Conjugate (495/515)	Invitrogen	Cat# L32481

Biological samples		

40 IPMN Patient Samples	Vanderbilt University Medical Center	N/A

Chemicals, peptides, and recombinant proteins		

Chitinase from *streptomyces griseus*	Sigma-Aldrich	Cat# C613
PNGaseF	New England Biolabs	Cat# P0704L
PNGase F Prime	Bulldog Bio	Cat# NZPP050LY
Growth factor reduced Matrigel	Corning	Cat# 356231
Lipofectamine RNAiMAX Transfection Reagent	Thermo Fisher Scientific	Cat# 13778075
RPMI 1640 with L-glutamine	Corning	Cat# 10-040-CV
Tet system approved, Fetal bovine serum	Gibco	Cat# A4736401
Doxycycline Hydrochloride	RPI Research Products	Cat# D43020
ProLong Gold Antifade Mountant with DAPI	Invitrogen	Cat# P36931
Advanced DMEM/F12	Gibco	Cat# 12634010
B-27 Supplement	Gibco	Cat# 17504044
Nicotinamide	Sigma-Aldrich	Cat# N0636
*N*-Acetyl-L-cysteine	Sigma-Aldrich	Cat# A9165
Immobilon Western Chemiluminescent HRP substrate	Milipore Sigma	Cat# WBKLS0500
Collagenase, Type IV, powder	Gibco	Cat# 17104019
Soybean trypsin i nhibitor, powder	Gibco	Cat# 17075029
Dispase	Stemcell Technologies	Cat# 07913
Recombinant mouse EGF	BioLegend	Cat# 585606
Recombinant mouse FGF-10	BioLegend	Cat# 751002
Gastrin-1, human	Anaspec	Cat# AS-20750
A 83-01	Tocris	Cat# 2939
Rock i nhibitor, Y-27632	HelloBio	Cat# HB2297
Mayer’s Hematoxylin Solution	Sigma-Aldrich	Cat# MHS32
Glycerol	Sigma-Aldrich	Cat# G5516

Critical commercial assays		

Pierce BCA Protein Assay	Thermo Fisher Scientific	Cat# 23225
Quick-RNA MiniPrep Kit	Zymo Research	Cat# R1055
Luna Universal One-Step RT-qPCR Kit	New England Biolabs	Cat# E3005
Corning BioCoat Matrigel Invasion Chambers	Corning	Cat# 354480
4-20% Mini-Protean TGX Precast Protein Gels	Bio-rad	Cat# 456103
AEC+ High Sensitivity Substrate Chromogen	Dako	Cat# K3469
Impact DAB substrate peroxidase (HRP)	Vector	Cat# SK-4105
Alcian blue PAS Stain Kit	Abcam	Cat# ab245876
Qubit RNA assay	Thermo Fisher Scientific	Cat# Q10210
NEBNext Poly(A) mRNA magnetic isolation module	New England Biolabs	Cat# E7490
NEBNext Ultra II directional RNA library prep	New England Biolabs	Cat# E7765L
KAPA Library quantification complete kit	Roche	Cat# KK873

Deposited data		

Pancreatic Adenocarcinoma (QCMG, Nature 2016)	cBioPortal	https://www.cbioportal.org/study/summary?id=paad_qcmg_uq_2016
Bulk Human RNA Sequencing data	Moffitt et al.^60^	GEO: GSE71729
Single Cell Murine Pancreatitis data	Ma et al.^13^	GEO: GSE172380
Bulk RNA-seq on 4838 cell line	This paper	GEO; GSE244280
Bulk RNA-seq on C241 cell line	This paper	GEO; GSE244280

Experimental models; Cell lines		

Mouse: 4838	Anirban Maitra	N/A
Mouse: C241	Anirban Maitra	N/A

Experimental models; Qrganisms/strains		

Mouse; *LSL-Kras^G12D/+^, Ptf1a^Cre/+^, LSL-rtTA-TetO-GNAS^R201C^*	Anirban Maitra	n/a

Oligonucleotides		

siRNA targeting Spdef	Thermo Fisher Scientific	Cat# 184152
siRNA targeting Creb3l1	Thermo Fisher Scientific	Cat# 162495
siRNA nonspecific control	Thermo Fisher Scientific	Cat# AM4613
Forward primer for quantitative PCR of Aqp5 GCC ATC TTG TGG GGA TCT AC	Custom oligo were made by IDT	n/a
Reverse primer for quantitative PCR of Aqp5 CCC AGA AGA CCC AGT GAG AG	Custom oligo were made by IDT	n/a
Forward primer for quantitative PCR of B4galnt3 ATCA GCC TCC CTC AGG TAC T	Custom oligo were made by IDT	n/a
Reverse primer for quantitative PCR of B4galnt3 AAC TTG GCT CCA GGG TCA TT	Custom oligo were made by IDT	n/a
Forward primer for quantitative PCR of Creb3l1 GAC CAA CGC AAC CAC TGT TC	Custom oligo were made by IDT	n/a
Reverse primer for quantitative PCR of Creb3l1 TAA GGG AGA GGT GAC GGG AG	Custom oligo were made by IDT	n/a
Forward primer for quantitative PCR of Epcam ATT TGC TCC AAA CTG GCG TCT	Custom oligo were made by IDT	n/a
Reverse primer for quantitative PCR of Epcam GTT GTT CTG GAT CGC CCC TT	Custom oligo were made by IDT	n/a
Forward primer for quantitative PCR of Fn1 GGG AGG AAG AAG ACA GAT GAG C	Custom oligo were made by IDT	n/a
Reverse primer for quantitative PCR of Fn1 TGA ACT GTG GAG GGA ACA TCC	Custom oligo were made by IDT	n/a
Forward primer for quantitative PCR of Gkn1 ACA ATG TTC GTC GTG GGT CT	Custom oligo were made by IDT	N/A
Reverse primer for quantitative PCR of Gkn1 ACT GCT GTC CAC TTC CGT CT	Custom oligo were made by IDT	N/A
Forward primer for quantitative PCR of GNAS CCT GAG TGT GAT GAA CGT GCC TG	Custom oligo were made by IDT	N/A
Reverse primer for quantitative PCR of GNAS CGA AGC AGG TCC TGA TCG CTC	Custom oligo were made by IDT	N/A
Forward primer for quantitative PCR of Mgat4a GTT CCA CAG CGG CAA TCA AG	Custom oligo were made by IDT	N/A
Reverse primer for quantitative PCR of Mgat4a CTG ATT TCC AAA CTG TCG CTC TTA	Custom oligo were made by IDT	N/A
Forward primer for quantitative PCR of Rplpo GCC AAT AAG GTG CCA GCT G	Custom oligo were made by IDT	N/A
Reverse primer for quantitative PCR of Rplpo CTC CCA CCT TGT CTC CAG TC	Custom oligo were made by IDT	N/A
Forward primer for quantitative PCR of Spdef TCC TCT CTG CTC ACT CTG AA	Custom oligo were made by IDT	N/A
Reverse primer for quantitative PCR of Spdef AGA GCT CAT GTG TAT CCC TAG A	Custom oligo were made by IDT	N/A
Forward primer for quantitative PCR of Zeb1 GCG GCG CAA TAA CGT TAC AAA	Custom oligo were made by IDT	N/A
Reverse primer for quantitative PCR of Zeb1 TGG TCT GCT GGC AGT TCA TC	Custom oligo were made by IDT	N/A

Software and algorithms		

Fiji	NIH^4^	https://imagej.net/ij/
QuPath v0.4.0	Bankhead et al.^21^	https://qupath.github.io
GraphPad Prism v9.2.0	Dotmatios	https://www.graphpad.com/
R studio v4.4.2	The Comprehensive R Archive Network	https://cran.r-project.org/
DESeq2 v1.34.0	Love et al.^17^	N/A
Seurat	Hao et al.^61^	N/A
ggplot2	Wickham et al.^62^	N/A
SeuratObject	Sajita et al.^63^	N/A
patchwork	Pedersen et al.^64^	N/A
harmony	Korsunsky et al.^65^	N/A
dplyr	Wickham et al.^66^	N/A
devools	Wickham et al.^67^	N/A
STAR v2.7.9a	Dobin et al.^68^	https://github.com/alexdobin/STAR
Matplotlib	Matpotlib	https://github.com/matplotlib/matplotlib
Scanpy	Wolf et al.^69^	https://github.com/theislab/scanpy
Spectronaut 19	Biognosys	N/A
MSstats	Choi et al.^70^	N/A
MSstatsConvert	Staniak et al.^71^	N/A
tidyr	Wickham et al.^72^	N/A
stringr	Wickham.^73^	N/A
pySCENIC	Aibar et al.^74^	https://github.com/aertslab/pySCENIC

Other		

Doxycycline Chow	Tusculum Feed Center	Cat# 9205-0827
Olympus VS200 Slide Scanner	Olympus	N/A
Orbitrap Eclipse Tribrid Mass Spectrometer	Thermo Fisher Scientific	N/A
Zeiss AxioScan Z1 Slide Scanner	Zeiss	N/A
NovaSeq 6000	Illumina	N/A

## Highlights

- Human IPMNs show pyloric-type metaplasia with expression of gastric SPEM markers
- Mutant GNAS^R201C^ amplifies a pyloric phenotype and promotes indolent pancreatic precancer
- GNAS-driven SPDEF and CREB3L1 regulate transcriptional programs of pyloric identity
- GNAS^R201C^ reprograms glycosylation to increase LacdiNAcs and reduce Lewis antigens
