# Supplementary material for: Mutant GNAS drives a pyloric metaplasia with tumor suppressive glycans in intraductal papillary mucinous neoplasia
Source: Cell Rep. Author manuscript; Available in PMC 2026 Jan 31. (PMC12860476; doi:10.1016/j.celrep.2025.116684)
Supplement: 1 [file NIHMS2132770-supplement-1.pdf]

Cell Reports, Volume *44*

Supplemental information

Mutant GNAS drives a pyloric metaplasia with tumor suppressive glycans in intraductal papillary mucinous neoplasia

Vincent Quoc-Huy Trinh, Katherine E. Ankenbauer, Sabrina M. Torbit, Christopher P. Taranto, Jiayue Liu, Maelle Batardiere, Bhoj Kumar, H. Carlo Maurer, Frank Revetta, Zhengyi Chen, Angela R.S. Kruse, Audra M. Judd, Celina Copeland, Jahg Wong, Olivia Ben-Levy, Brenda Jarvis, Monica Brown, Jeffrey W. Brown, Koushik Das, Yuki Makino, Jeffrey M. Spraggins, Ken S. Lau, Parastoo Azadi, Anirban Maitra, Marcus C.B. Tan, and Kathleen E. DelGiorno

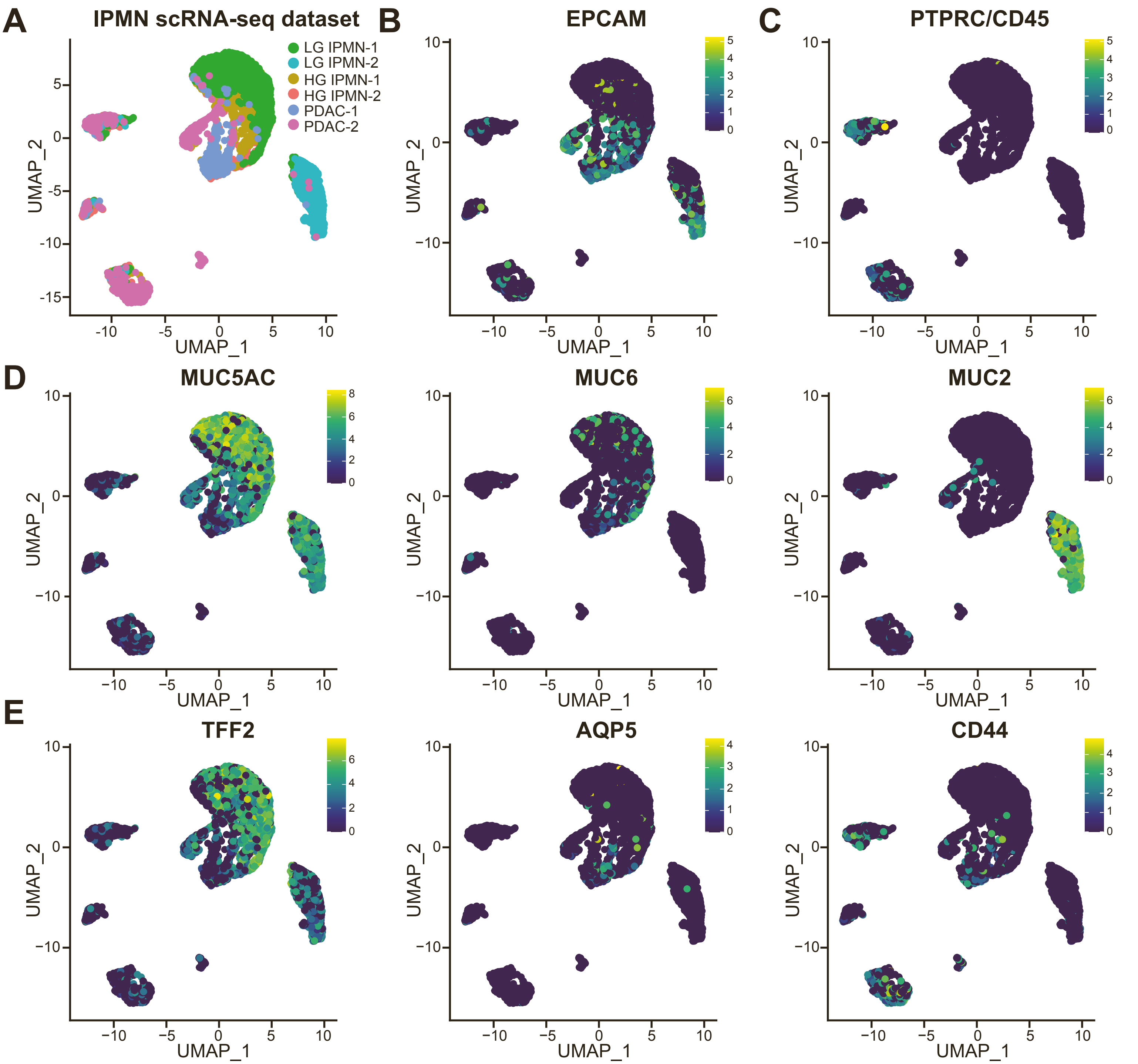

Figure S1. Expression of pyloric metaplasia markers in human IPMN. (**A**) Uniform manifold approximation and projection (UMAP) of single cell RNA-sequencing (scRNA-seq) from 6 patients with IPMN and/or PDAC. (**B**) Expression of epithelial marker *EPCAM* and (**C**) immune marker *PTPRC* (CD45). (**D**) Expression of mucins *MUC5AC*, *MUC6* (gastric folveolar IPMN), and *MUC2* (intestinal IPMN). (**E**) Expression of SPEM markers *TFF2*, *AQP5*, and *CD44*. Related to Figure 1.

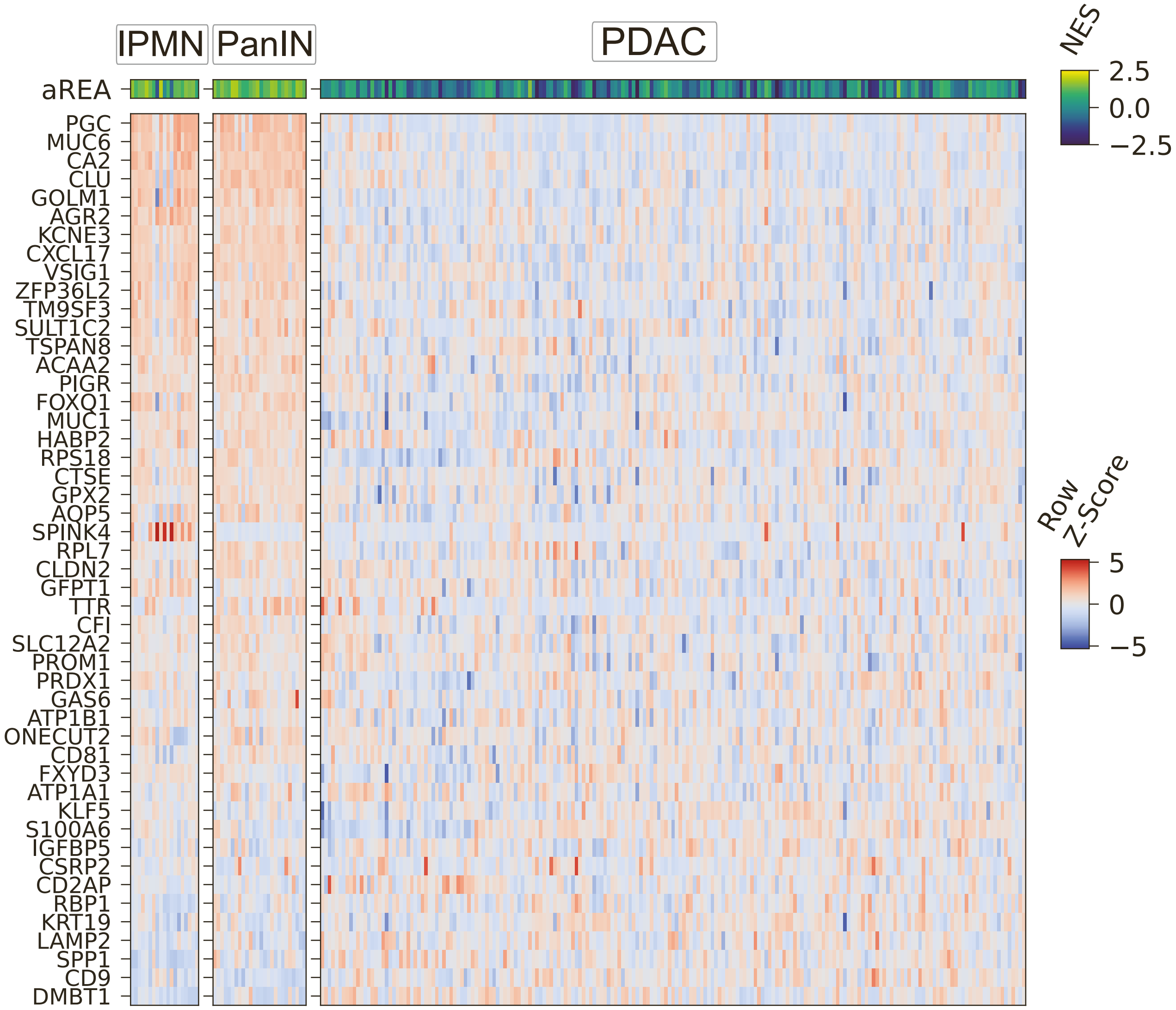

Figure S2. Expression of pyloric metaplasia markers in human pre-malignant lesions and PDAC. Heat map showing expression of SPEM markers identified in a murine model of pancreatitis in a previously reported dataset of laser capture dissected epithelium from patient IPMN (n = 19), PanIN (n = 26), and PDAC (n = 197)[20,21]. Related to Figure 1.

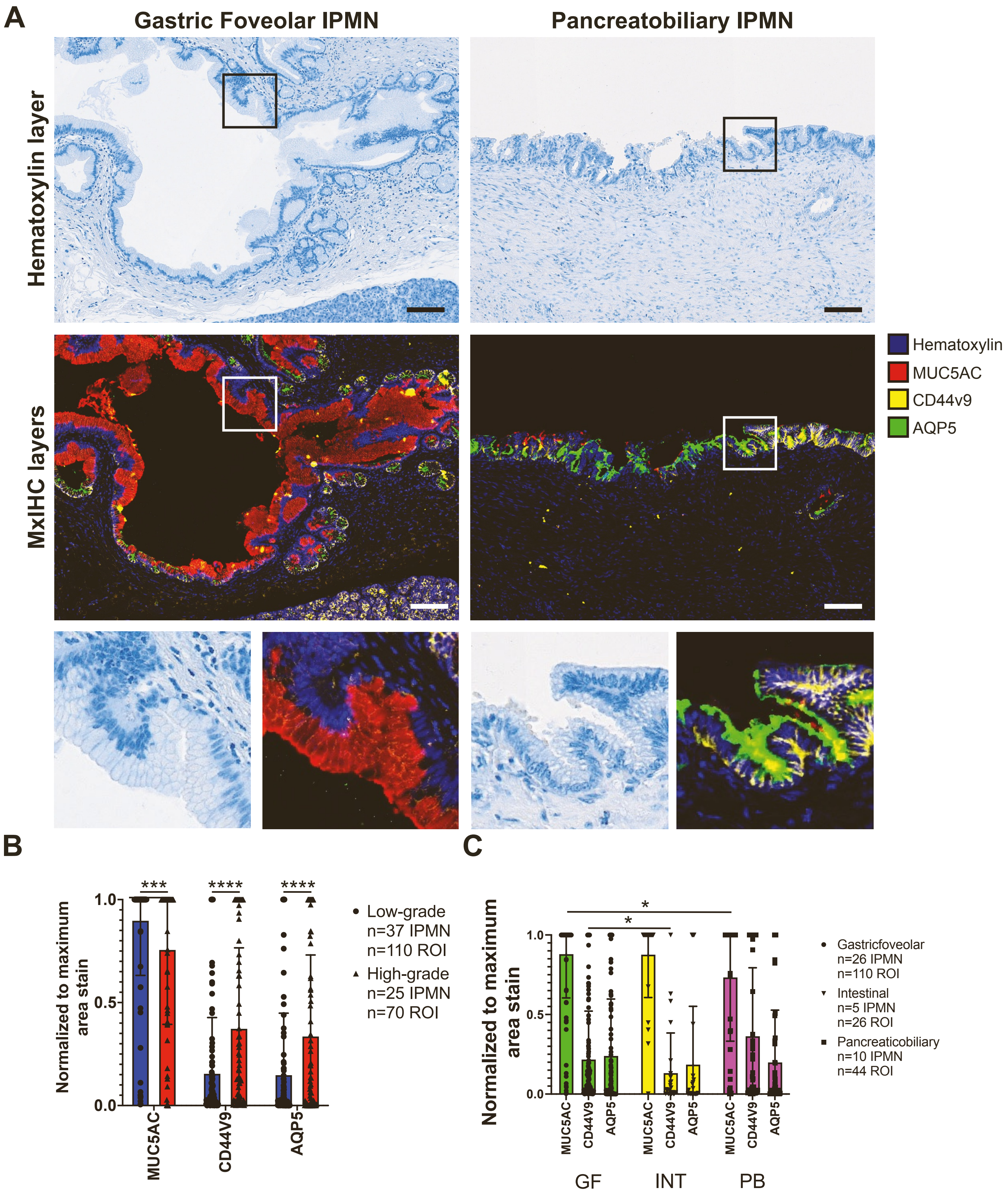

Figure S3. Pyloric metaplasia markers are expressed in human IPMN. (**A**) Representative images from hematoxylin, MUC5AC, CD44v9, and AQP5 from MxIHC separated by low grade gastric foveolar IPMN or high grade pancreatobiliary IPMN. Quantification of staining in (A) and Figure 2 plotted as (**B**) low grade vs. high grade or (**C**) by molecular subtype (gastric foveloar, intestinal, or pancreatobiliary). Scale bars, 100 μm. *, $p < 0.05$; ***, $p < 0.005$, ****, $p < 0.001$. Related to Figure 1.

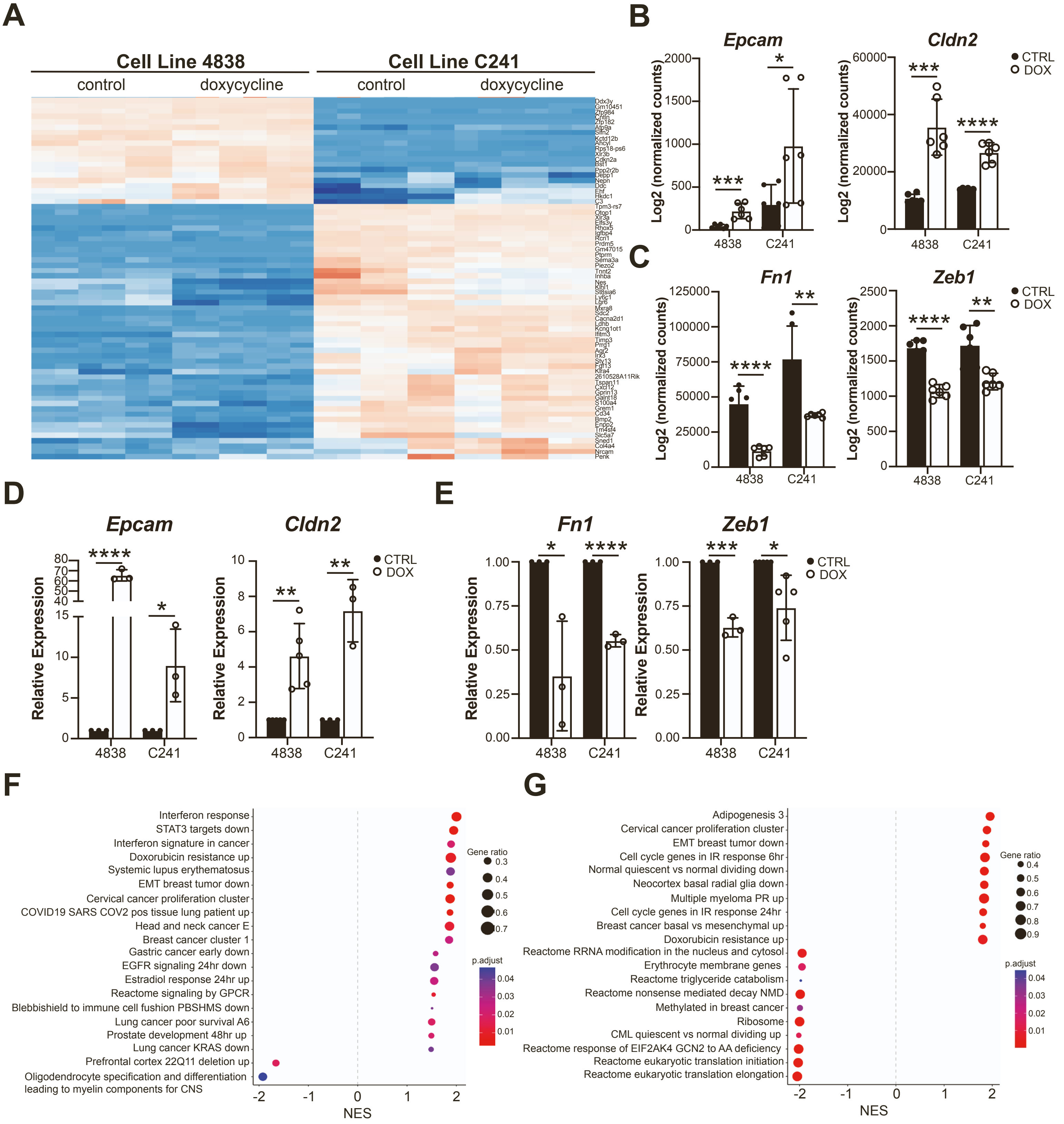

Figure S4. GNAS^{R201C} expression drives transcriptomic changes in PDAC cell lines. (**A**) Heat map of differentially expressed genes between cell lines 4838 and C241. (**B**) Expression of epithelial markers or (**C**) mesenchymal markers in 4838 or C241 cells +/- DOX treatment determined by RNA sequencing. (**D**) Expression of epithelial markers or (**E**) mesenchymal markers in 4838 or C241 cells +/- DOX treatment determined by qRT-PCR. (**F**) GSEA analysis of gene expression signature changes in 4838 or (**G**) C241 cells with DOX treatment. CTRL, control, black circles; DOX, doxycycline, white circles.*, $p < 0.05$; **, $p < 0.01$; ***, $p < 0.005$; ****, $p < 0.001$. Related to Figure 2.

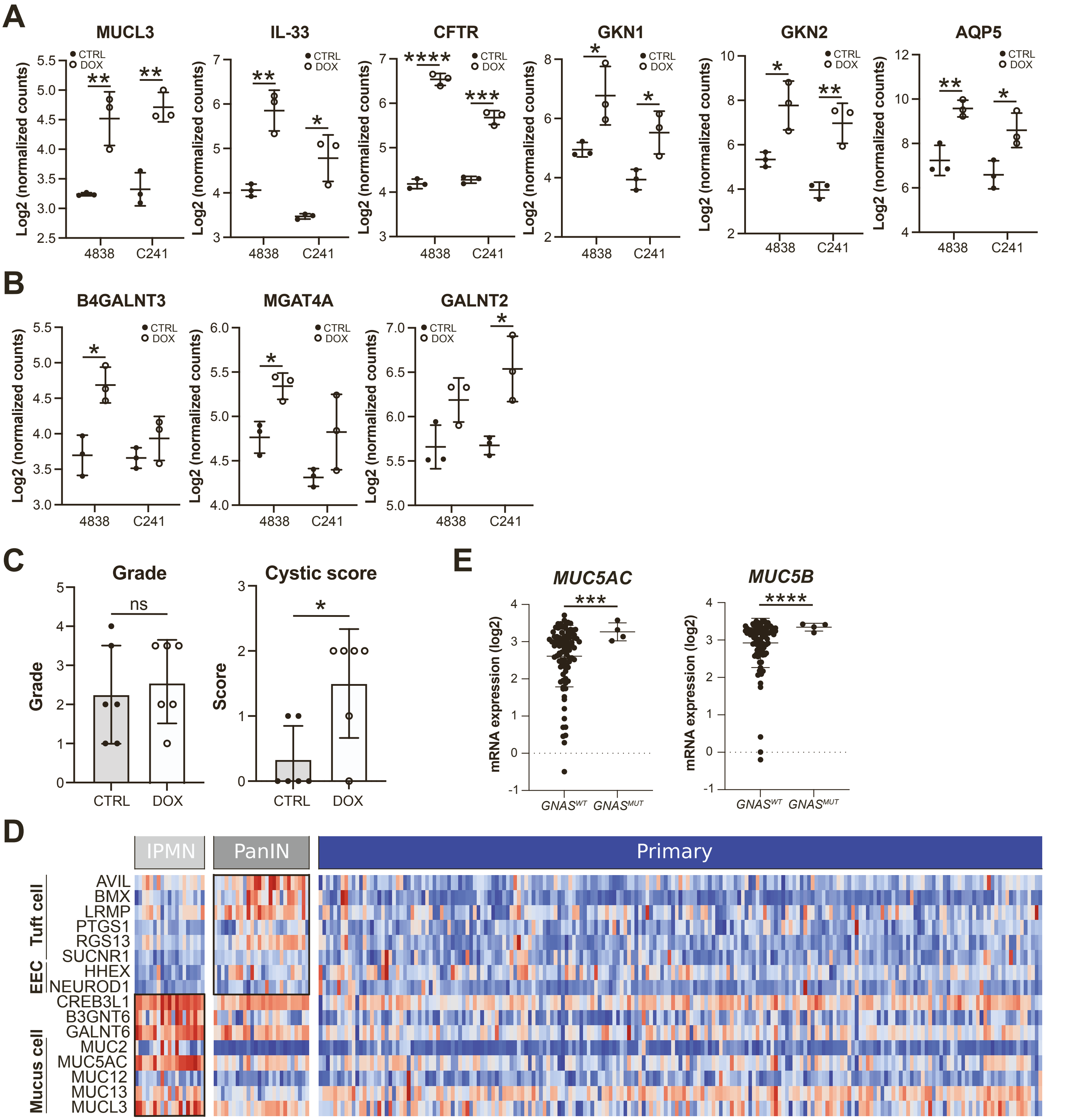

Figure S5. GNAS^{R201C} expression drives a mucinous, cystic phenotype. (**A**) Quantification of select pyloric or (**B**) glycosyltransferase protein abundance by mass spectrometry in 4838 or C241 cell lines treated +/- DOX. (**C**) Quantification of highest-grade lesion or cystic score in *Kras;GNAS* mice +/- DOX (combined 10- and 20-weeks treatment). Grade, 1 = 1/3 of tissue is low grade; 2 = 2/3 of tissue is low grade; 3 = high grade; 4 = invasive. Cystic score, 0 = no cysts; 1 = mild; 2 = extensive. (**D**) Heat map showing expression of select tuft cell, enteroendocrine cell (EEC), or mucus cell gene markers from Maurer et al.,[20,21] from the epithelium of human IPMN (n = 19), PanIN (n = 26), or PDAC (n = 197). (**E**) *MUC5AC* or *MUC5B* expression in human PDAC tumors from cBioPortal with wild type (n = 92) or mutant GNAS$^{R201C/H}$ (n = 4). *, p < 0.05; **, p < 0.01; ***, p < 0.005; ****, p < 0.001. Related to Figure 2.

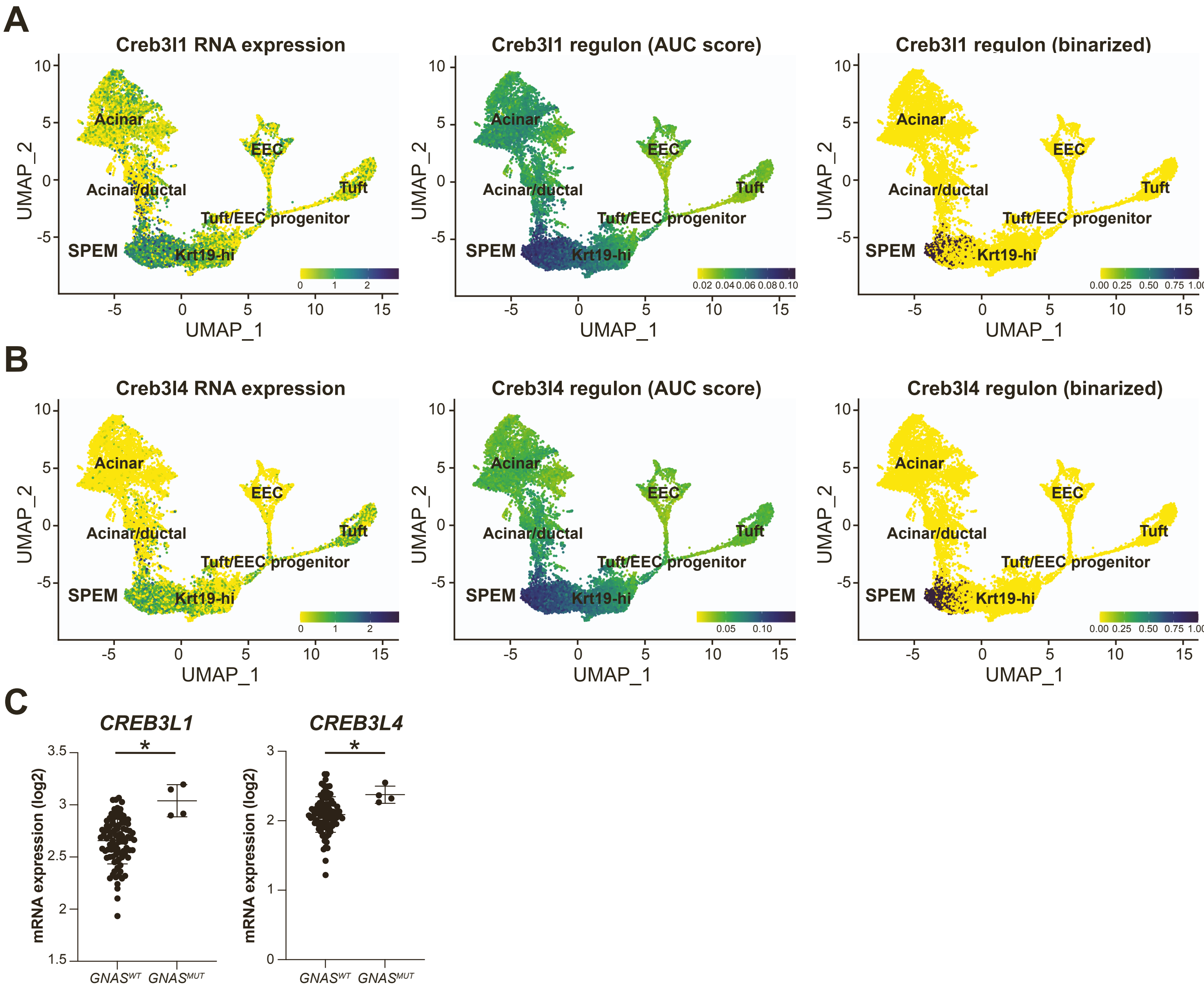

Figure S6. *Creb3l1* and *Creb3l4* are predicted regulators of SPEM. RNA expression or binarized activity of (**A**) *Creb3l1* or (**B**) *Creb3l4* predicted by Regulon analysis overlayed on the UMAP from Figure 3A. (**C**) Expression of *CREB3L1* or *CREB3L4* in human PDAC tumors from cBioPortal expressing either wild type (n = 96) or mutant *GNAS* (n = 4). *, $p < 0.05$. Related to Figure 3.

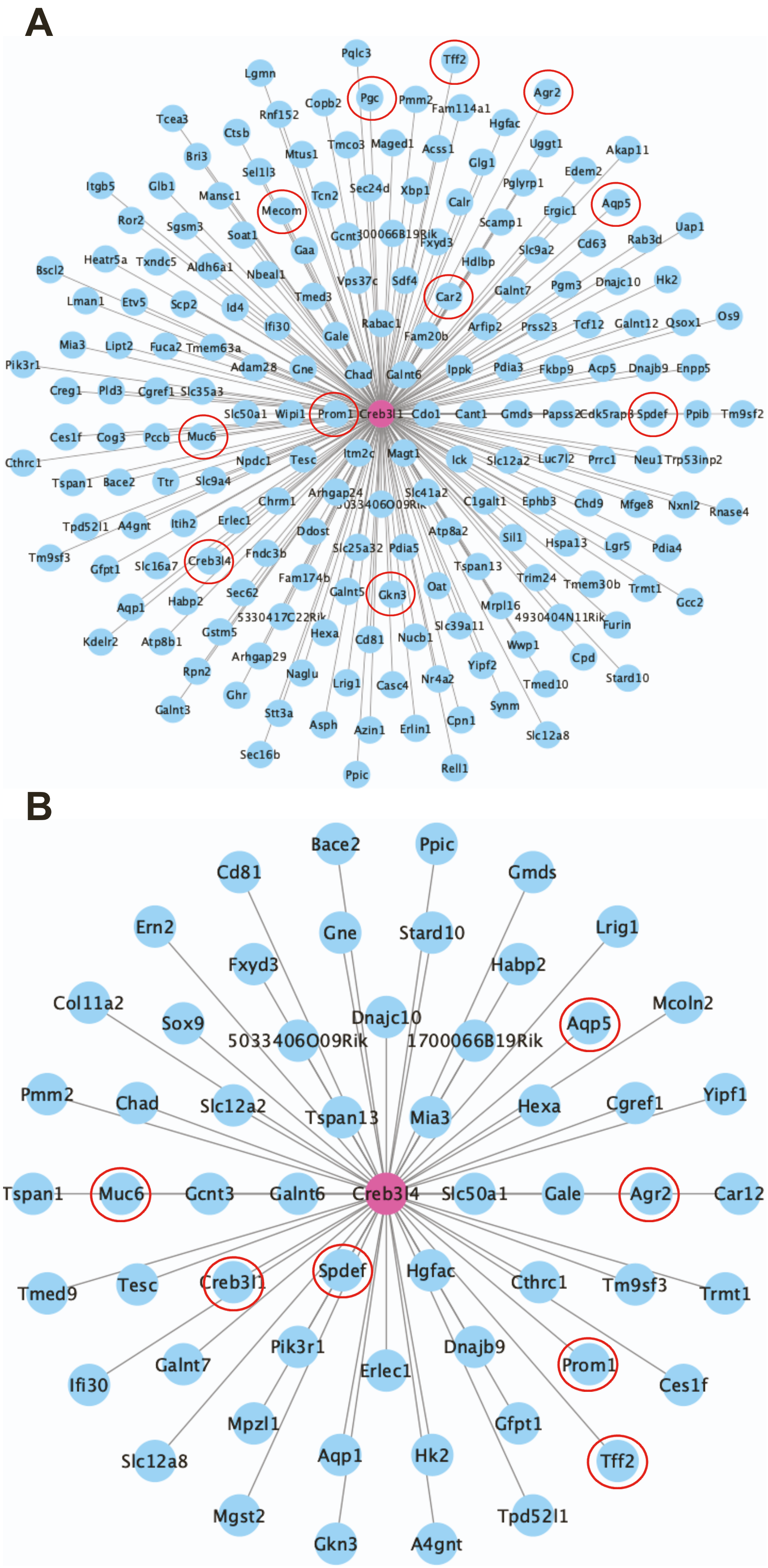

Figure S7. Creb3l1 and Creb3l4 are predicted regulators of SPEM. Plots of either (**A**) Creb3l1 or (**B**) Creb3l4 predicted target genes generated from PyScenic Regulon analysis of scRNA-seq data generated from murine pancreatitis. Select markers are circled in red. Related to Figure 3.

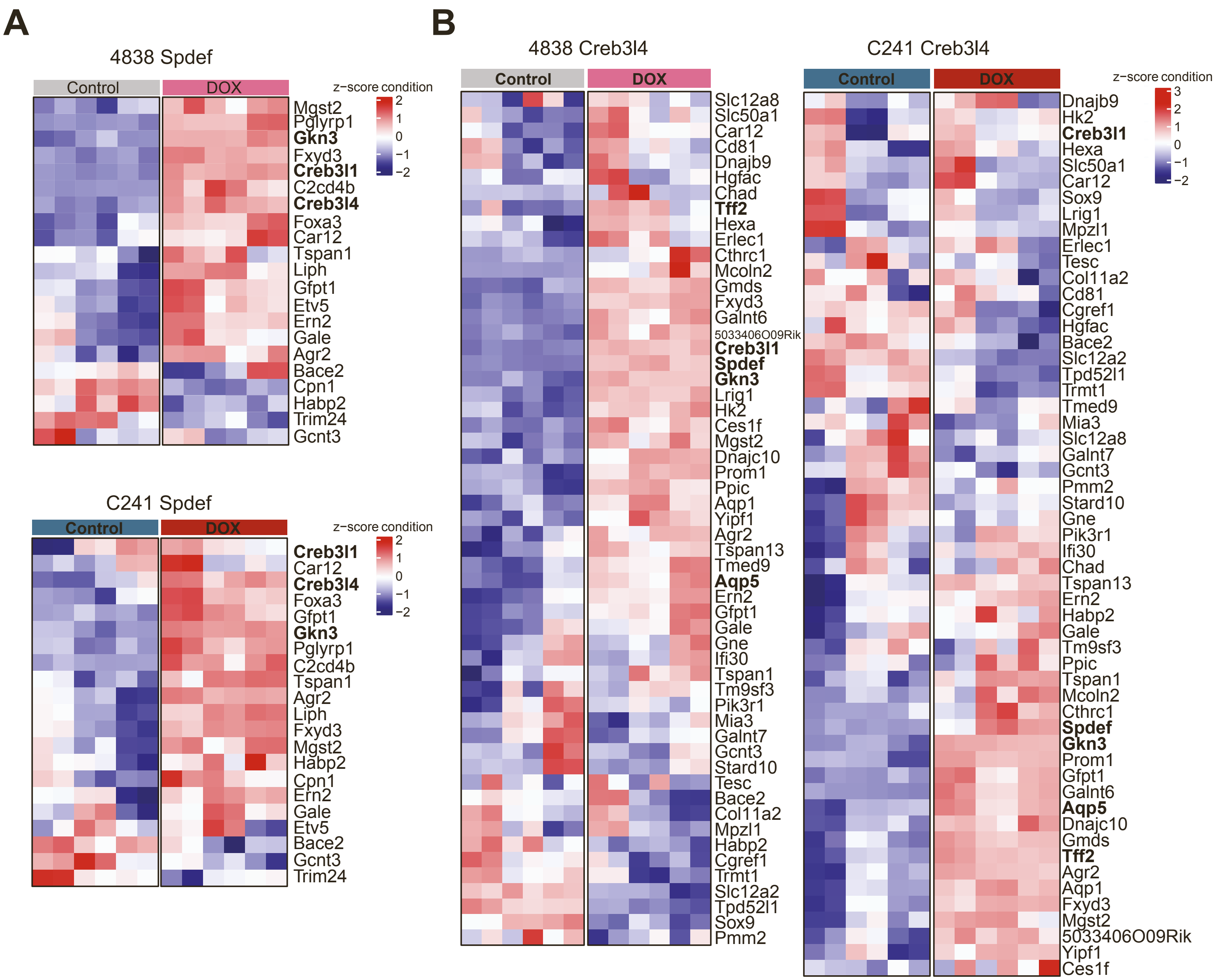

Figure S8. Spdef and Creb3l4 target genes increase with GNAS^{R201C} expression. Heat maps of (**A**) *Spdef* target genes overlaid on RNA-seq data of 4838 or C241 cells +/- DOX and *GNAS^{R201C}* expression or (**B**) *Creb3l4* target genes overlaid on RNA-seq data of either 4838 (left) or C241 (right) cells +/- DOX. Top 10% of gene targets are shown. Select SPEM markers are bolded. Related to Figure 3.

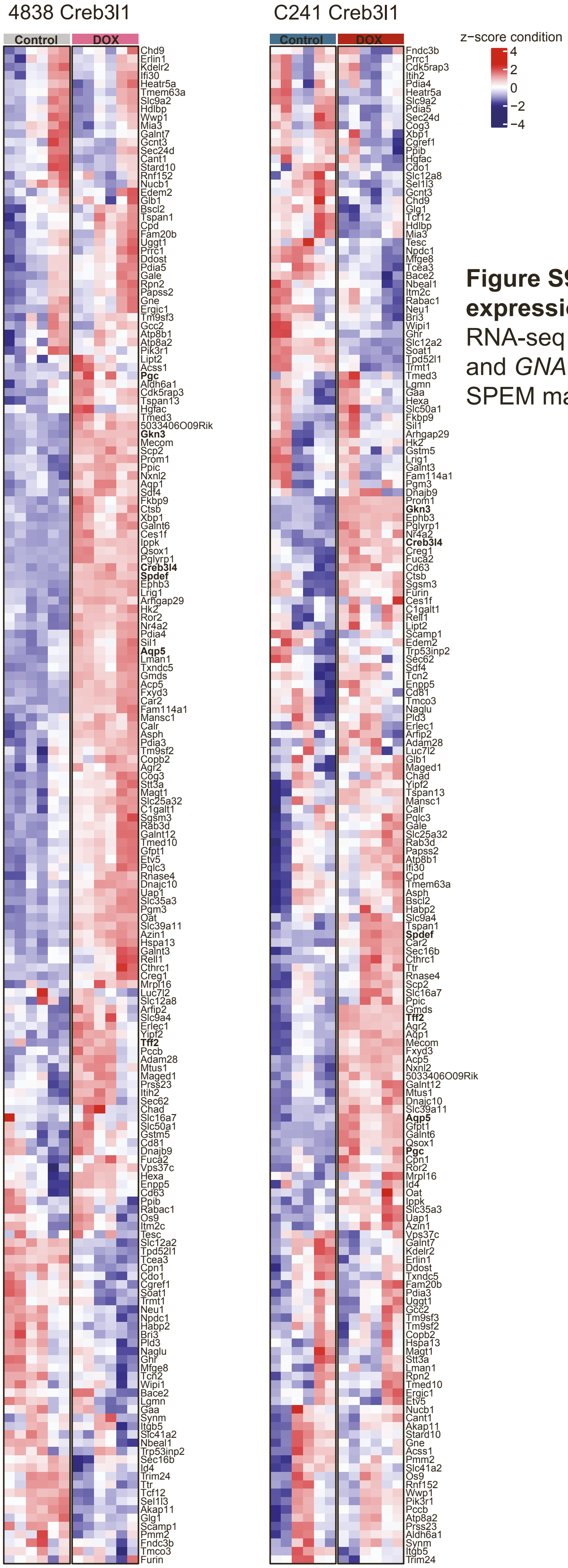

Figure S9. Creb3l1 target genes increase with GNAS^{R201C} expression. Heat maps of *Creb3l1* target genes overlaid on RNA-seq data of either 4838 (left) or C241 (right) cells +/- DOX and *GNAS^{R201C}* expression. Top 10% of gene targets are shown. SPEM markers are highlighted. Related to Figure 3.

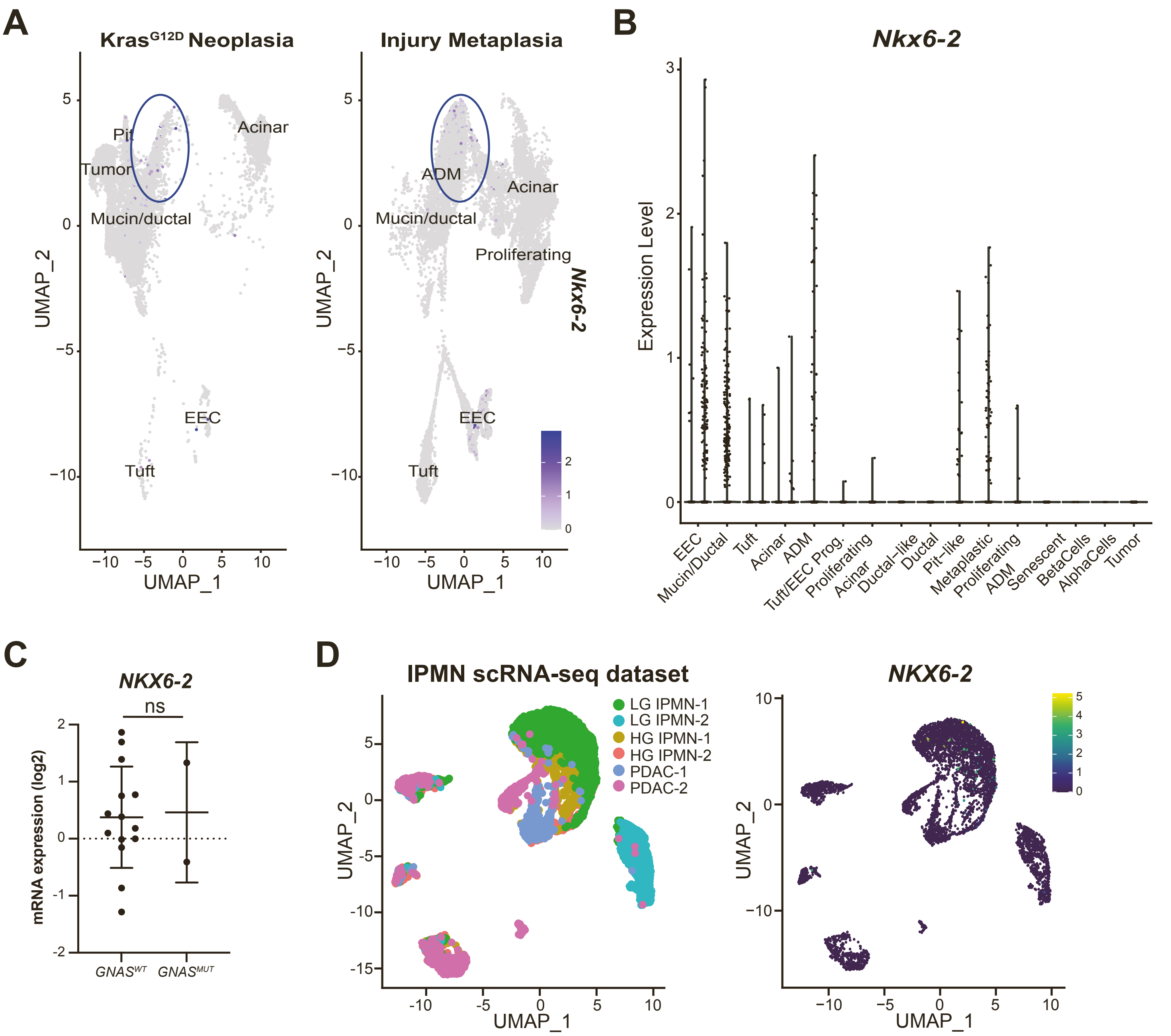

Figure S10. Expression of transcription factor NKX6-2 in murine and patient RNA-seq datasets.
(**A**) UMAP or (**B**) Violin plot of FastMNN integrated datasets from an injury-induced metaplasia scRNAseq dataset and a *Kras*^{G12D}-induced dataset of neoplasia and cancer[13,14]. (**C**) Expression of *NKX6-2* in human PDAC tumors from cBioPortal expressing either wild type (n = 14/92) or mutant *GNAS* (n = 2/4). (**D**) UMAP of scRNA-seq from human IPMN from Figure S1 with *NKX6-2* expression[18]. Related to Figure 3.

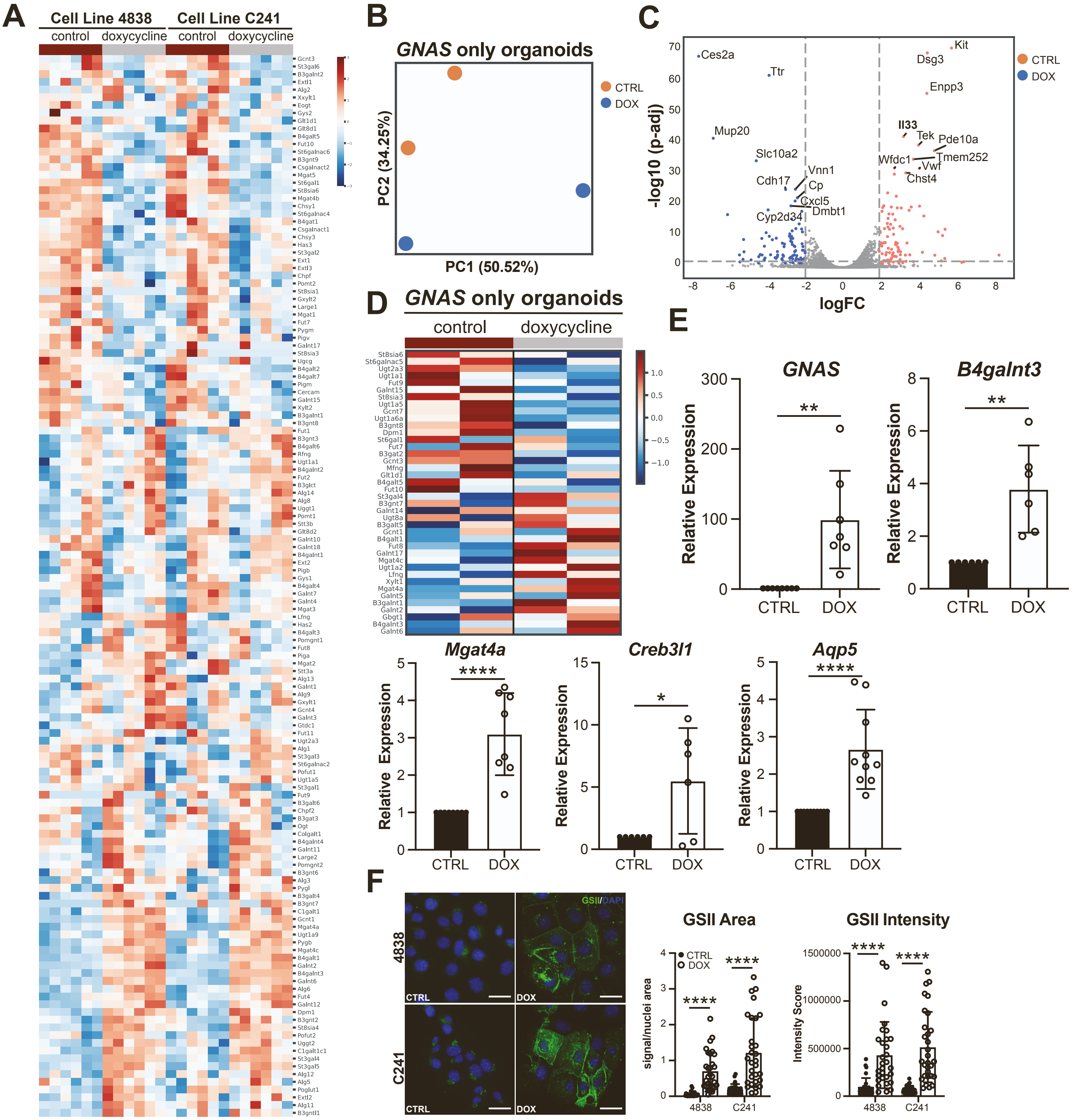

Figure S11. GNAS^{R201C} drives glycosyltransferase gene expression changes. (**A**) Heat map of glycosyltransferase gene expression in either 4838 or C241 cells treated +/- DOX. (**B**) PCA of RNA-seq generated from organoids from *Ptf1a$^{Cre/+}$;Rosa26R-LSL-rtTA-TetO-Gnas^{R201C}* (*GNAS*) mice treated with either control or DOX *in vitro* (n = 2 biological replicates/condition). (**C**) Volcano plot of differentially expressed genes between control and DOX treated groups. (**D**) Heat map of glycosyltransferase gene expression in *GNAS* organoids treated +/- DOX. (**E**) qRT-PCR for human *GNAS*, *B4galnt3*, *Mgat4a*, *Creb3l1*, or *Aqp5* in *GNAS* only organoids +/- DOX. (**F**) IF and quantification of lectin GSII (green) recognizing GlcNAcs and DAPI (blue) in 4838 and C214 cells +/- DOX. Scale bars, 50 μm. *, $p < 0.05$; **, $p < 0.01$; ****, $p < 0.001$. Related to Figure 4.

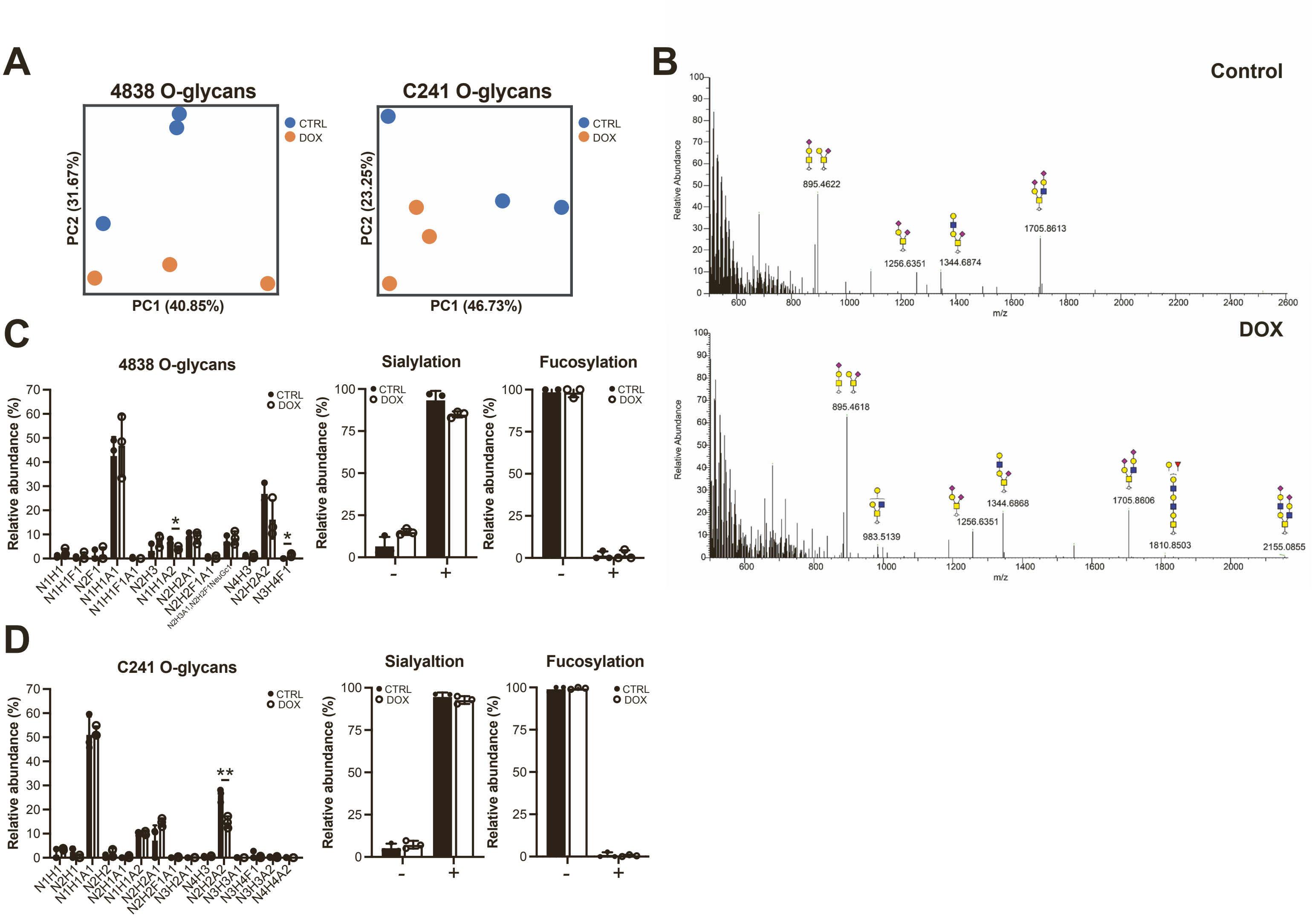

Figure S12. GNAS^{R201C} expression drives minor O-glycan changes. (**A**) Principal component analysis of total O-glycan species changes between control and DOX treated 4838 or C241 cells. (**B**) Representative O-glycosylation mass spectrometry spectra in C241 cells; control, top, and DOX-treated, bottom. (**C**) O-glycans and modifications identified in 4838 cells +/- DOX. (**D**) O-glycans and modifications identified in C241 cells +/- DOX. *, $p < 0.05$; **, $p < 0.01$. Related to Figure 5.

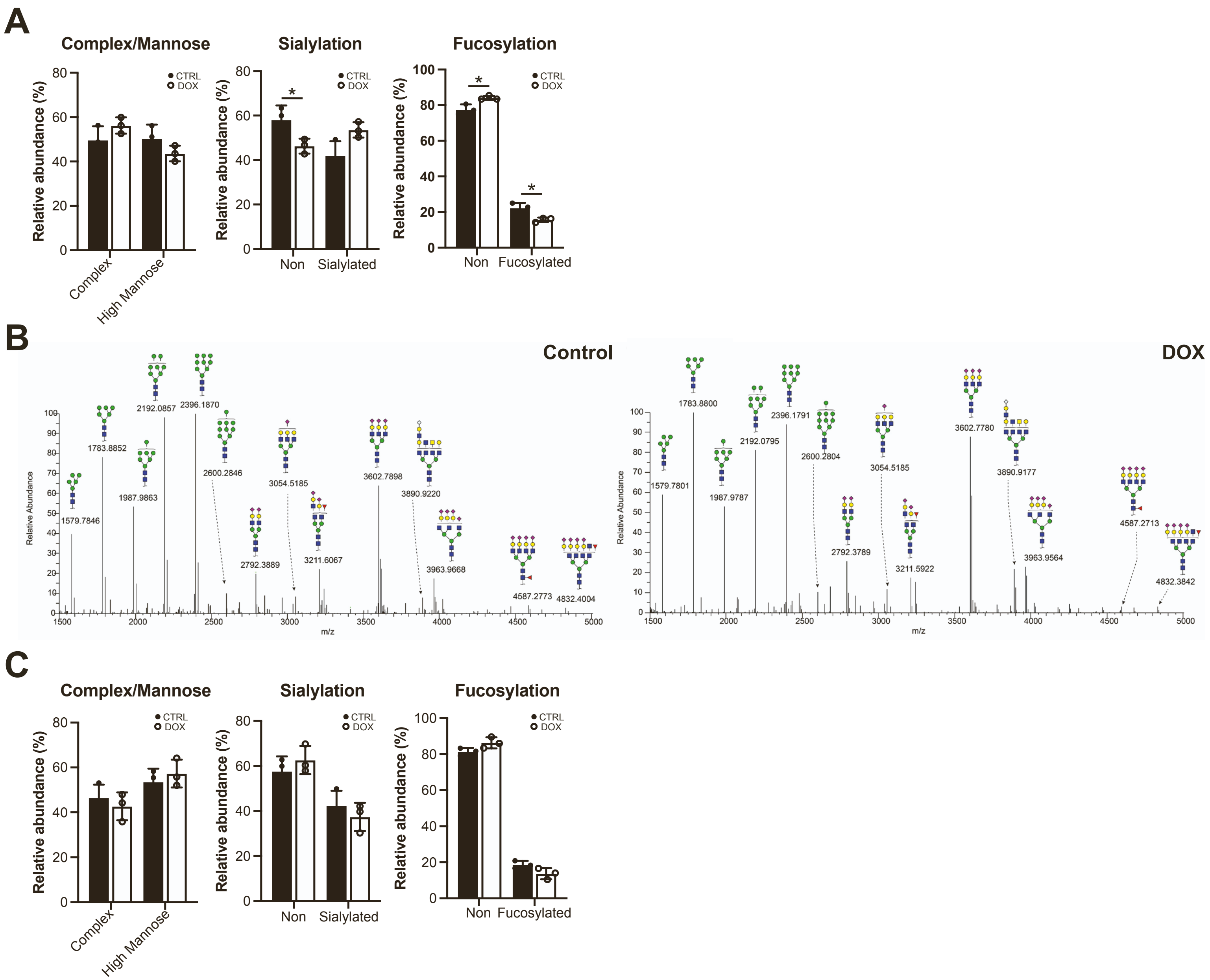

Figure S13. GNAS^{R201C} expression drives major N-glycan changes. (**A**) N-glycan modifications identified in 4838 cells +/- DOX. (**B**) N-glycosylation mass spectrometry spectra; control, left and DOX-treated, right and (**C**) N-glycan modifications identified in C241 cells +/- DOX. *, $p < 0.05$. Related to Figure 5.

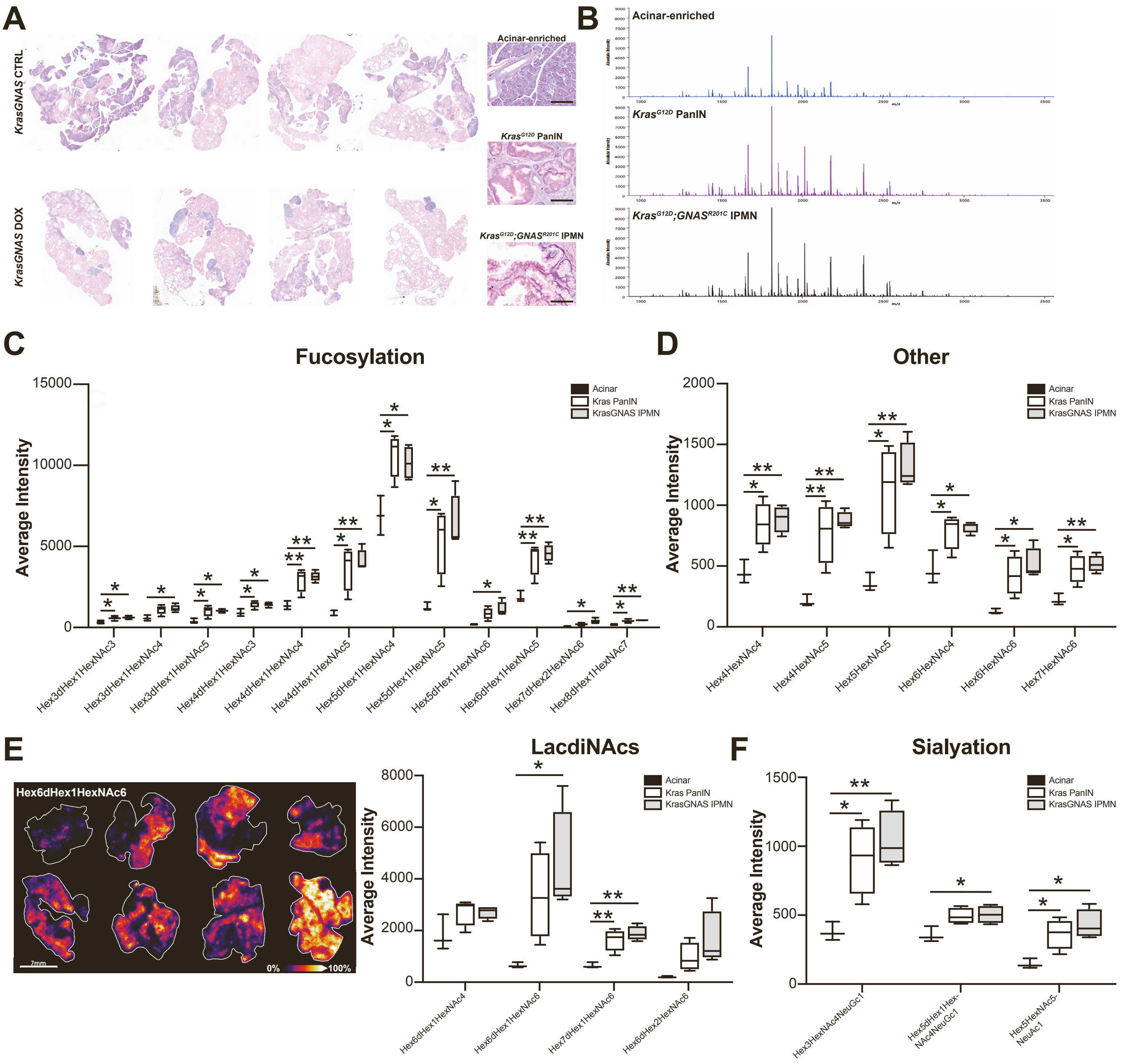

Figure S14. PanIN and IPMN formation are accompanied by significant changes in glycan deposition. (**A**) Hematoxylin and eosin (H&E) staining of pancreata from *KrasGNAS* mice on either control or DOX chow for 22 weeks. Scale bars, 100 μm. (**B**) Spectra collected by imaging glycosylation mass spectrometry of acinar enriched areas or PanIN from *KrasGNAS* mice on control chow or IPMN enriched areas from *KrasGNAS* mice on DOX chow. (**C**) Quantification of predicted glycans characterized by fucosylation or (**D**) additional glycans. (**E**) Representative heat map of expression of a predicted LacdiNAc (control tissues, top; DOX chow, bottom) and quantification of identified species. Scale bar, 7 mm. (**F**) Quantification of predicted glycan structures characterized by sialyation. *, $p < 0.05$; **, $p < 0.01$. Related to Figure 5.

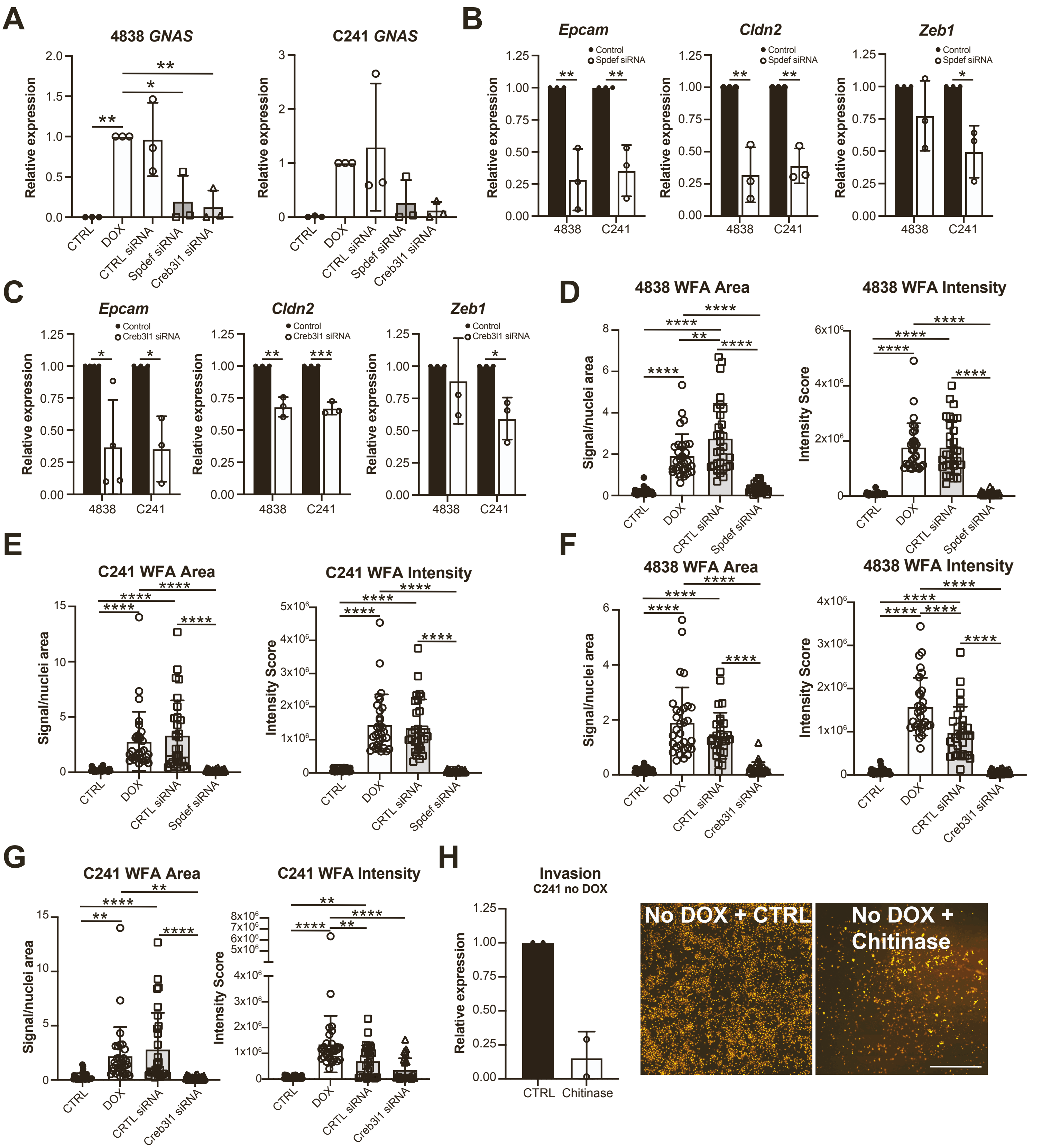

Figure S15. SPDEF, CREB3L1 and Chitinase impact cell phenotype and LacdiNAc abundance. (**A**) qRT-PCR for human *GNAS* in either 4838 or C421 cells treated with either control (CTRL), doxycycline (DOX) or DOX+ control siRNA or DOX+siRNAs against *Spdef* or *Creb3l1*. (**B**) qRT-PCR for *Epcam*, *Cldn2*, or *Zeb1* in 4838 or C241 cells treated with either DOX + control siRNA or siRNAs against *Spdef* or (**C**) *Creb3l1*. (**D**) Quantification of lectin WFA (LacdiNAcs) area and signal intensity in 4838 or (**E**) C241 cells treated with DOX and either control or *Spdef* siRNA. (**F**) Quantification of WFA area and signal intensity in 4838 or (**G**) C241 cells treated with DOX and either control or *Creb3l1* siRNA. (**H**) Quantification and representative images of invasion for C241 cells treated with either control or chitinase. Scale bar, 1mm. *, $p < 0.05$; **, $p < 0.01$; ***, $p < 0.005$; ****, $p < 0.001$. Related to Figure 6.

Table S1. Demographic data of IPMN patients. Related to STAR Methods.

Slide	Grade	Main Variant	Age at time of surgery	Sex	Surgical procedure/ Operation	Race	History of Diabetes	
S02-18519	HG	INT	69	M	Whipple (2002)	White	No	IPMN-2, PB
S04-01058	LG	GF	63	F	Whipple (2004)	White	Yes	IPMN-2, GF (BRANCH)
S05-00053	LG	INT	71	M	Whipple (2005)	White	No	IPMN-2, GF+PB
S05-12465	LG	GF	55	M	Whipple (2005)	White	Yes	IPMN-2-GF (BRANCH)
S05-16811	LG	PB	64	M	Whipple (2005)	White	No	IPMN-2-GF (MAIN)
S05-26331	LG	PB	67	F	Whipple (2005)	White	No	IPMN-2-GF
S06-03104	HG	PB	72	M	Whipple (2006)	White	No	IPMN-3-GF-PB (BRANCH)
S06-14228	LG	GF	61	F	Whipple (2006)	White	No	IPMN-2-PB-GF (MAIN)
S06-15082	LG	INT	81	M	Whipple (2006)	White	No	IPMN-3-PB
S06-28504	LG	GF	52	M	Whipple (2006)	White	No	IPMN-3 PB+GF(MAIN)
S07-11282	LG	GF	70	F	Whipple (2007)	White	No	IPMN-2 PB (MAIN+SIDE)
S08-12147	HG	GF	46	F	Whipple (2008)	White	Yes	IPMN-3 FOCAL IPMN (MAIN)
S08-29792	LG	GF	84	M	Whipple (2008)	White	No	IPMN-GF-2
S08-30564	LG	GF	61	F	Whipple (2008)	Black	No	IPMN-2-GF
S09-34420	HG	INT	57	M	Whipple (2009)	White	No	IPMN-2-PB-GF (MAIN)
S10-02105	LG	GF	75	F	Laproscopic Distal Pancreatetectomy (2010)	White	No	IPMN1-2
S10-02333	LG	INT	80	M	Whipple (2010)	White	No	IPMN
S10-11830	LG	PB	53	F	Distal Pancreatetectomy (2010)	White	No	POSSIBLE RETENSION CYST
S10-16223	LG	GF	63	F	Whipple (2010)	White	No	RETENSION CYST
S10-21194	LG	GF	78	M	Pancreatetectomy (2013)	White	No	IPMN-2 VERSUS RETENSION CYST
S10-31747	LG	GF	76	M	Pancreatetectomy with splenectomy (2010)	White	Yes	PDAC IN IPMN-GF-2
S10-33961	LG	GF	79	F	Pancreatectomy with splenectomy and removal of foreign body (2010)	White	No	IPMN-PB-2
S11-00385	LG	GF	75	F	Whipple (2011)	Black	No	IPMN-PB-2
S11-02963	LG	GF	73	F	Pancreatectomy with splenectomy and cholecystectomy (2011)	White	Yes	IPMN
S11-37118	LG	GF	71	M	Whipple (2011)	White	No	IPMN
S11-03866	LG	GF	59	F	Pancreatetectomy with splenectomy and gastrostomy (2011)	White	Yes	RETENSION CYST AND IPMN-2-GF
S11-06505	LG	GF	54	F	Whipple (2011)	White	Yes	RETENSION CYST
S11-07329	HG	GF	59	F	Whipple (2011); Anastomosis extrahepatic biliary ducts; benign lesion excision	Black	No	G3 PDAC arising from IPMN
S11-19160	HG	GF	62	M	Distal Pancreatectomy (2011)	Black	Yes	PDAC IN IPMN-PB-3
S12-07444	HG	PB	80	M	Puestow (Pancreaticojejunostomy) (2012)	White	No	IPMN3
S12-29195	LG	GF	53	M	Distal Pancreatetectomy (2012)	White	Yes	IPMN3
S12-30184	HG	PB	53	M	Whipple (2012)	White	No	PDAC from main IPMN3
S13-17835	HG	PB	65	F	Whipple (2013)	White	Yes	COLLOID CA from IPMN with HG
S13-35969	LG	GF	76	F	Whipple (2013)	White	No	IPMN, LG
S14-10297	LG	GF	76	F	Laproscopic Distal Pancreatetectomy with Cystoscopy (2014)	White	No	PDAC from IPMN
S14-21643	HG	PB	68	M	Whipple (2014)	White	No	mucinous (colloid) from IPMN
S15-01111	LG	INT	70	M	Whipple; incisional herniorraphy (2015)	White	Yes	IPMN, LG
S16-23918	LG	PB	67	M	Whipple (2016)	White	No	PDAC from IPMN
S16-37214	LG	GF	75	F	Whipple; laparoscopic revision/removal gastric band; splenectomy (2016)	White	Yes	PDAC from IPMN
S17-24838	LG	GF	69	M	Whipple (2017)	White, Native Hawaiian or Other Pacific Islander	No	IPMN, High Grade
